# Differential contribution for ERK1 and ERK2 kinases in BRAF^V600E^-triggered phenotypes in adult mouse models

**DOI:** 10.1038/s41418-024-01300-x

**Published:** 2024-05-02

**Authors:** Giuseppe Bosso, Ana Carolina Cintra Herpst, Oscar Laguía, Sarah Adetchessi, Rosa Serrano, Maria A. Blasco

**Affiliations:** Telomeres and Telomerase Group, Molecular Oncology Program, Spanish National Cancer Centre (CNIO), Melchor Fernández Almagro 3, Madrid, E-28029 Spain

**Keywords:** Cancer genetics, Lymphocytes, Cell biology, Lung cancer

## Abstract

The BRAF gene is mutated in a plethora of human cancers. The majority of such molecular lesions result in the expression of a constitutively active BRAF variant (BRAF^V600E^) which continuously bolsters cell proliferation. Although we recently addressed the early effects triggered by BRAF^V600E^-activation, the specific contribution of ERK1 and ERK2 in BRAF^V600E^-driven responses in vivo has never been explored. Here we describe the first murine model suitable for genetically dissecting the ERK1/ERK2 impact in multiple phenotypes induced by ubiquitous BRAF^V600E^-expression. We unveil that ERK1 is dispensable for BRAF^V600E^-dependent lifespan shortening and for BRAF^V600E^-driven tumor growth. We show that BRAF^V600E^-expression provokes an ERK1-independent lymphocyte depletion which does not rely on p21^CIP1^-induced cell cycle arrest and is unresponsive to ERK-chemical inhibition. Moreover, we also reveal that ERK1 is dispensable for BRAF^V600E^-triggered cytotoxicity in lungs and that ERK-chemical inhibition abrogates some of these detrimental effects, such as DNA damage, in Club cells but not in pulmonary lymphocytes. Our data suggest that ERK1/ERK2 contribution to BRAF^V600E^-driven phenotypes is dynamic and varies dependently on cell type, the biological function, and the level of ERK-pathway activation. Our findings also provide useful insights into the comprehension of BRAF^V600E^-driven malignancies pathophysiology as well as the consequences in vivo of novel ERK pathway-targeted anti-cancer therapies.

## Introduction

The BRAF gene, coding for a master kinase of RAS-RAF-MEK-ERK (ERK-) pathway, is mutated in a plethora of human cancers [1–5]. The vast majority of such molecular lesions affect codon 600 of the corresponding gene product, and out of these, ~90% is represented by the 1799T>A nucleotide transition resulting in the substitution of glutamic acid for valine. The resulting BRAF^V600E^ kinase is regarded as a constitutively active BRAF [6] mutant which bolsters cell proliferation independently of external mitogenic stimuli.

A consistent number of genetically engineered mouse models in which BRAF^V600E^ expression is driven by tissue-specific promoters shed light on the role of this oncogene in human malignancies [7–14]. Nevertheless, such a strategy did not allow to dissect the overall impact in vivo of the early phenotypes elicited by ubiquitous BRAF^V600E^-activation. Albeit we recently addressed such point [15], the specific contribution of ERK1 and ERK2 in the BRAF^V600E^-driven responses [15] in vivo, such as reduced lifespan, tumor initiation and progression, oncogene-induced senescence and cytotoxicity in different cell types such as Club Cells (CCs) and lymphocytes has never been explored. Here we generated the very first murine model which allows to genetically dissect the contribution of ERK1/ERK2 in multiple phenotypes induced by chronic as well as acute ubiquitous BRAF^V600E^-expression.

## Results

### Role of ERK1 in BRAF^V600E^ mouse viability and in the development of spontaneous BRAF^V600E^-driven tumors

**Fig. 1:**
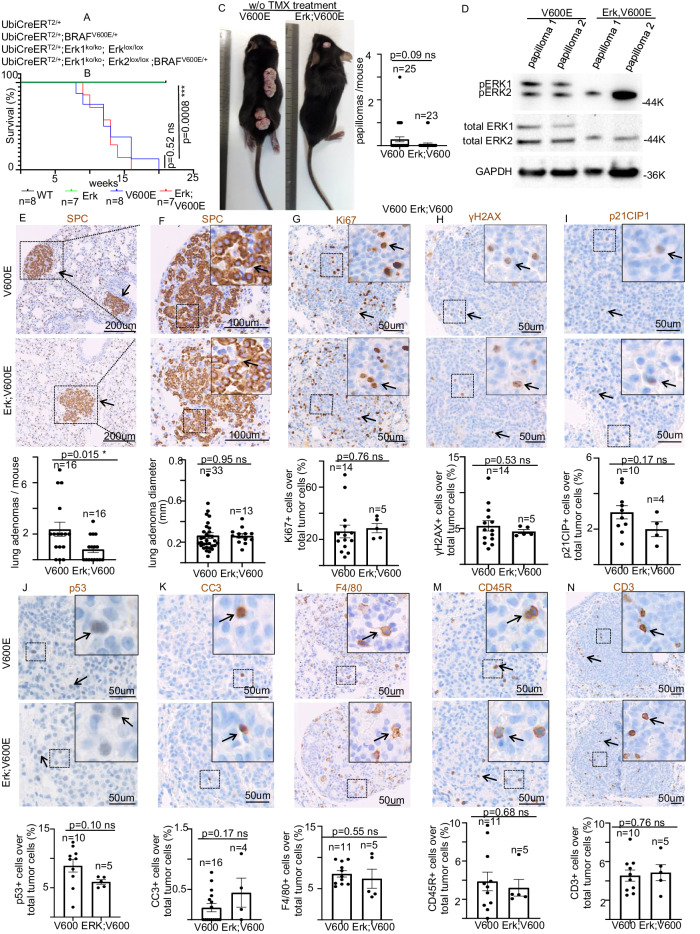
Role of ERK1 in BRAF^V600E^ mouse viability and in the development of spontaneous BRAF^V600E^-driven tumors. **A** Schematic representation of mouse genotypes employed in this study. **B** Survival curves of mice with the indicated genotype. n= animals per group. ***P < 0.001. (Log Rank test). **C** Representative images (left) and quantification (right) of the number of BRAF^V600E^-driven papillomas in BRAF^V600E^ and Erk, BRAF^V600E^ mice. **D** Representative images showing immunoblot experiments in papilloma protein extracts from BRAF^V600E^ and Erk, BRAF^V600E^ mice. GAPDH is used as a loading control. Representative images (top) and quantifications showing (bottom) **E**, **F** SPC, **G** Ki67, **H** γH2AX, (**I**) p21^CIP1^, **J** p53, **K** Cleaved Caspase 3 (CC3), **L** F4/80, **M** CD45R, **N** CD3 immunostainings in lung sections from BRAF^V600E^ and Erk, BRAF^V600E^ mice. Quantifications were performed on at least five different lung adenomas from at least five different mice per experimental group. Data are expressed as mean ± SEM; *n* = tumors per group. *P < 0.05; ns not significant. (T-Student’s test unpaired). Arrows point to selected positive cells for the indicated marker. Insets: magnifications of areas inside dashed squares. The arrow in (**E**) points to an SPC+ lung adenoma.

To establish the role of ERK1 and ERK2 in the BRAF^V600E^-induced phenotypes in vivo [15], we generated UbiCreER^T2/+^; Erk1^−/−^; Erk2^lox/lox^; BRAF^LSL_V600E/+^ (Erk;BRAF^V600E^) mice harboring UbiCreER^T2^ allele [15, 16], expressing the conditionally active CreER^T2^ recombinase gene under the control of the human ubiquitin promoter (UbiCreER^T2^), combined with BRAF^LSL_V600E^ allele [8, 15] (BRAF^V600E^), the conditional floxed allele for Erk2 [17] (Erk2^lox/lox^) and the constitutive knock-out allele for Erk1 [18] (Erk1^−/−^). As control groups we used the mice bearing the UbiCreER^T2/+^ allele alone (wild-type; WT) or in combination with either Erk1^−/−^; Erk2^lox/lox^ alleles (Erk) or BRAF^LSL_V600E/+^ (BRAF^V600E^) (Fig. 1A).

We recently provided evidence that the spontaneous Cre-mediated activation of BRAF^V600E^ over time resulted in reduced viability, development of spontaneous skin papillomas and lung adenomas in mouse models [15] in the absence of tamoxifen (TMX) treatment for the Cre activation. Interestingly, in a way similar to the BRAF^V600E^ mice, untreated 10-week-old Erk;BRAF^V600E^ mice, but not either the Erk or WT mice, showed weight loss, bad shape, papillomatous skin lesions and all of them died between 11 and 17 weeks from birth, thus suggesting that the Erk1 constitutive loss is not able to rescue the lethality depending on the random expression of BRAF^V600E^ due to spontaneous Cre-activation over time (Fig. 1B). Moreover, regardless the presence of ERK1, both BRAF^V600E^ and Erk;BRAF^V600E^ mice, but not either WT or Erk groups (Supplementary Fig. 1A), showed skin papillomas, due to spontaneous Cre-dependent recombination of BRAF^V600E^ allele (Supplementary Fig. 1B). Importantly, albeit Erk;BRAF^V600E^ mice tend to develop less papillomas, such a difference in tumor occurrence does not reach the statistical significance (Fig. 1C). Moreover, western blot (WB) analysis of such papillomatous lesions confirmed the total absence of ERK1, but not of ERK2, in Erk;BRAF^V600E^ papillomas compared to BRAF^V600E^ lesions (Fig. 1D), therefore strongly indicating that ERK1 constitutive abrogation does not prevent BRAF^V600E^ random activation from giving rise to spontaneous papillomas. However, the low frequency of spontaneous papillomas in both BRAF^V600E^ and Erk, BRAF^V600E^ mice prevented us from performing more detailed analyses in such skin tumors.

In contrast, although the constitutive ablation of ERK1 does not abolish the ability of BRAF^V600E^ to induce lung tumors arising from alveolar type II cells (ATIIs), compared to the BRAF^V600E^ strain, untreated Erk;BRAF^V600E^ mice showed a significant decrease in the number lung adenomas staining positive for the prosurfactant C (SPC) ATII marker (Fig. 1E; Supplementary Fig. 1C). WB experiments from the lungs of these mice revealed that, whereas neither total nor the phosphorylated fraction of ERK2 was altered, ERK1 signal was not detectable (Supplementary Fig. 1D). In line with this, Erk;BRAF^V600E^ adenomas display a dramatic decrease in the intensity of total ERK1/2 staining compared to BRAF^V600E^ tumors, thus further confirming that they have risen in an Erk1^−/−^ genetic background (Supplementary Fig. 2A). Importantly, upon Erk1 deletion, no significant changes in either tumor size, proliferative index, DNA damage, cell cycle arrest, senescence or apoptosis as indicated, respectively, by Ki67, γH2AX, p21^CIP1^, p53 and cleaved caspase 3 (CC3) markers, were observed in Erk;BRAF^V600E^ lung tumors compared to BRAF^V600E^ adenomas (Fig. 1E–K), thus strongly suggesting that ERK1 is dispensable for BRAF^V600E^-driven lung adenomas growth and progression. Moreover, by staining such adenomas with specific antibodies raised against F4/80, CD45R, and CD3, which are cell-specific markers respectively for macrophages, B-lymphocytes [19, 20], and T-lymphocytes [21, 22], we found no alterations in the recruitment of tumor-associated leukocytes (TALs) such as macrophages, T- and B- lymphocytes infiltrated inside the adenomas (Fig. 1L–N), therefore indicating that ERK1 loss does not affect the ability of BRAF^V600E^-driven tumors to recruit TALs. These findings suggest that, despite ERK1 loss may partially inhibit BRAF^V600E^ lung adenoma initiation, once the tumor is established, ERK1 does not affect either BRAF^V600E^-driven tumor development or immune cell recruitment to the adenomas. However, we do not rule out the possibility that a random inactivation of Erk2, in Erk1^−/−^ background, might play a role in such inhibitory effect in tumor initiation.

Next, to better understand how Erk1 genetic ablation might affect tumor initiation in Erk;BRAF^V600E^ strain, we also checked whether the lung parenchyma of such mice might display typical features of cell cytotoxicity. Nevertheless, histopathological analysis showed that the reduction in tumor number observed upon Erk1 loss is not associated to any alteration in the number of either Ki67-, p21^CIP1^- or CC3-positive cells in the alveolar parenchyma of Erk;BRAF^V600E^ mice compared to BRAF^V600E^ strain. Thus, these data suggest that the decreased adenoma occurrence observed in Erk;BRAF^V600E^ strain may not be ascribable to any detectable alteration in respectively cell proliferation, apoptosis or cell cycle arrest in the lung parenchyma (Supplementary Fig. 2B–D). Taken together these findings provide evidence that Erk1 kinase depletion is not sufficient to rescue either the BRAF^V600E^-dependent lethality or BRAF^V600E^-driven tumor growth.

### Acute BRAF^V600E^ activation provokes an overall lymphocyte depletion which is not rescued upon ERK1 loss

**Fig. 2:**
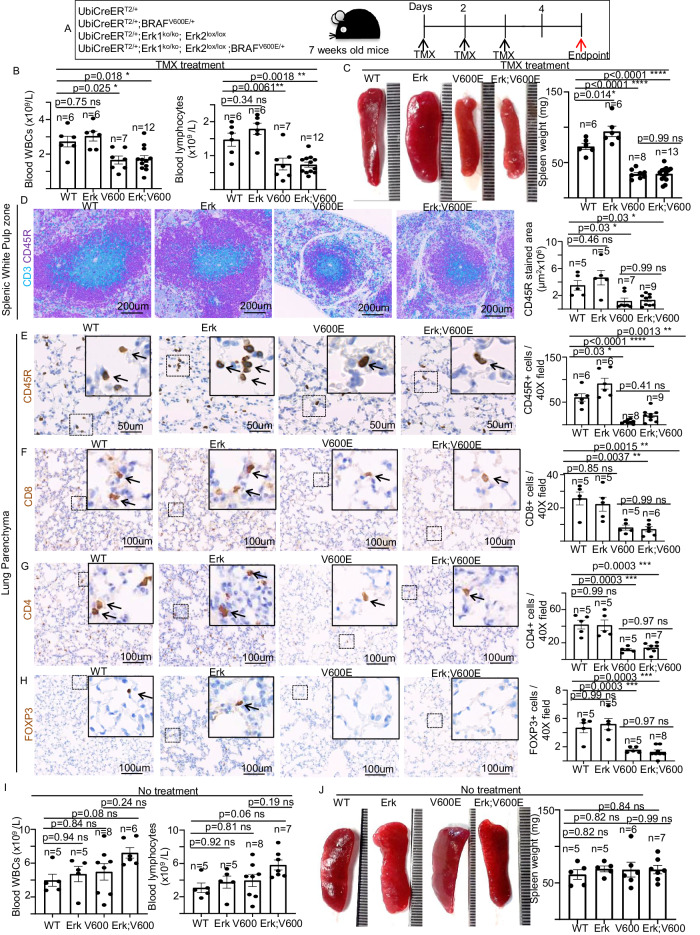
Acute conditional BRAF^V600E^ activation results in an overall lymphocyte depletion in adult mice which is not rescued upon ERK1 loss. **A** Schematic representation of mouse genotypes and experimental procedure employed in this study. TMX = 4-hydroxy tamoxifen; **B** Charts showing the number of total circulating white blood cells (left) and lymphocytes (right) of the indicated experimental groups upon TMX treatment. **C** Representative images and quantifications showing the spleen weight in the indicated experimental groups following TMX treatment. **D** Representative images and quantifications showing double CD3 CD45R immunostainings in spleen sections from WT, Erk, BRAF^V600E^, and Erk;BRAF^V600E^ mice. Representative images and quantifications showing **E** CD45R, **F** CD8, **G** CD4, and **H** FOXP3 immunostainings in lung sections of WT, Erk, BRAF^V600E^ and Erk;BRAF^V600E^ mice. **I** Charts showing the number of total circulating white blood cells (left) and lymphocytes (right) of the indicated experimental groups in the absence of TMX treatment. **J** Representative images and quantifications showing the spleen weight of the indicated experimental groups in the absence of TMX treatment. Quantifications were performed on at least five different areas of the sections in a random way. Data are expressed as mean ± SEM; *n* = animals per group. *P < 0.05; **P < 0.01; ***P < 0.001, ****P < 0.0001, ns not significant. (ANOVA test with Tukey’s post-hoc correction). Arrows point to selected positive cells for the indicated marker. Insets: magnifications of areas inside dashed squares.

To elicit an acute BRAF^V600E^ expression, 7-week-old mice were administered TMX intraperitoneally. As previously described [15], the BRAF^V600E^ strain, but neither the WT nor the Erk mice, started to appear sick 2–3 days post injection, displayed rapid weight loss, and needed to be sacrificed after 3–5 days (Fig. 2A, Supplementary Fig. 3A). However, 15 days after TMX treatment also Erk mice, but not the WT strain, started to die, confirming the evidence that the ubiquitous depletion of ERK1/2 kinases is lethal in adult mice [17]. Intriguingly, the BRAF^V600E^ and Erk;BRAF^V600E^ mice showed undistinguishable viability phenotypes upon TMX administration, suggesting that BRAF^V600E^ is epistatic over the constitutive Erk1 loss and the Erk2 conditional depletion. These findings also suggest that Erk1 lack is not able either to rescue or ameliorate the acute BRAF^V600E^-induced sickness, including the immediate weight loss and the early lethality (Supplementary Fig. 3A, B). PCR experiments confirmed Cre-dependent rearrangement of BRAF^V600E^ allele upon TMX treatment, resulting in the effective BRAF^V600E^ activation (Supplementary Fig. 3C). Interestingly, BRAF^V600E^ activation also resulted in a drastic decrease in both the number of circulating lymphocytes, but not other white blood cells (WBCs) (Supplementary Fig. 3D), and in spleen size compared to Erk and WT strains (Fig. 2B, C). Furthermore, double immunostainings for the detection of CD3+ and CD45R+ lymphocytes confirmed that the BRAF^V600E^-induced spleen size reduction was ascribable, at least in part, to the depletion of both B- and T-lymphocytes (Fig. 2D, Supplementary Fig. 3E). Importantly, such an acute blood and splenic lymphocyte depletion cannot be restored by the constitutive Erk1 loss, thus suggesting that Erk1 is dispensable for BRAF^V600E^-driven acute lymphocyte toxicity (Fig. 2B–D). Similarly, regardless the presence of Erk1, BRAF^V600E^-challenged mice also display a drastic decrease in the number of lung B-, T-helper, T-cytotoxic and T-regulatory lymphocytes, as revealed by the corresponding markers, CD45R, CD4 [23, 24], CD8 [25, 26] and FOXP3 [27, 28], thus further supporting the notion that BRAF^V600E^ results in an overall depletion of lymphocytes in an Erk1-independent fashion (Fig. 2E–H). Importantly, blood and lung lymphocyte depauperation, as well as spleen weight reduction, are not observed in age-matched untreated mice harboring the same genotypes, thus suggesting that such phenotypes are specifically ascribable to an acute, but not chronic, BRAF^V600E^ expression and occur within few days after TMX treatment (Fig. 2I, J, Supplementary Fig. 4A, B). Altogether, these data strongly suggest that ERK1 is dispensable for the rapid BRAF^V600E^-mediated general lymphocyte loss.

### Genetic and molecular dissection of ERK1/2 role in BRAF^V600E^-dependent cell toxicity in splenic lymphocytes

**Fig. 3:**
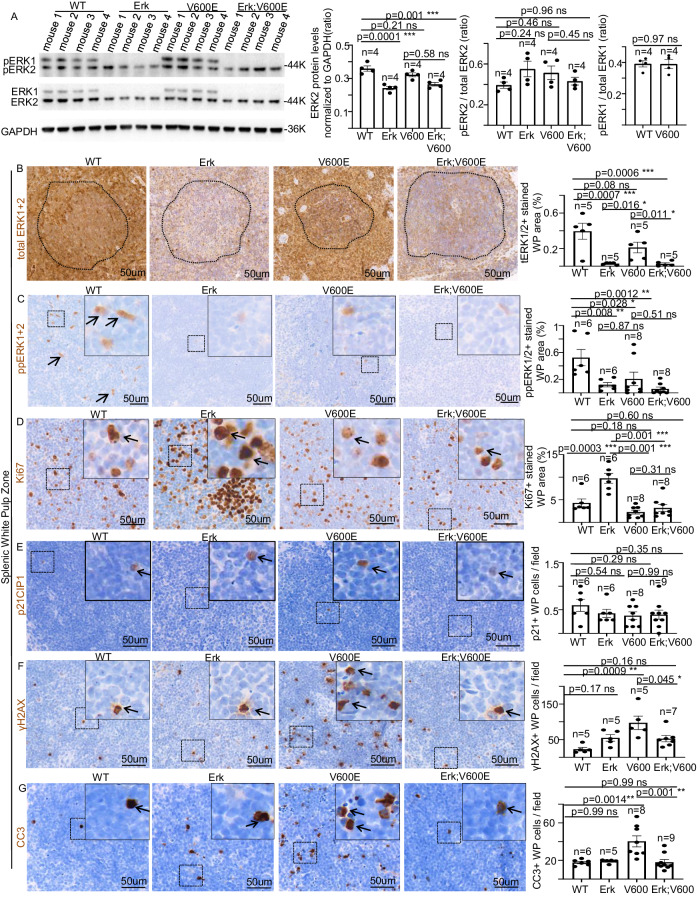
Genetic and molecular dissection of the role of ERK1/2 in BRAF^V600E^-dependent cell toxicity in splenic white pulp cells. **A** Representative images (left) and quantifications (right) showing immunoblot experiments in spleen protein extracts from WT, Erk, BRAF^V600E^, and Erk, BRAF^V600E^ mice. Data are expressed as mean ± SEM; *n* = animals per group. ***P < 0.001, ns = not significant. (ANOVA test with Tukey’s post-hoc correction). GAPDH is used as a loading control. Representative images and quantifications showing **B** total ERK1/2, **C** phosphorylated ERK1/2, **D** Ki67, **E** p21^CIP1^, **F** γH2AX and **G** CC3 immunostainings in spleen sections of WT, Erk, BRAF^V600E^ and Erk;BRAF^V600E^ mice. Quantifications were performed on at least five different areas of the sections in a random way. Data are expressed as mean ± SEM; *n* = animals per group. *P < 0.05; **P < 0.01; ***P < 0.001, ns not significant. (ANOVA test with Tukey’s post-hoc correction). Arrows point to selected positive cells for the indicated marker. Insets: magnifications of areas inside dashed squares. The dashed line in (**B**) marks the boundary between white and red pulps.

**Fig. 4:**
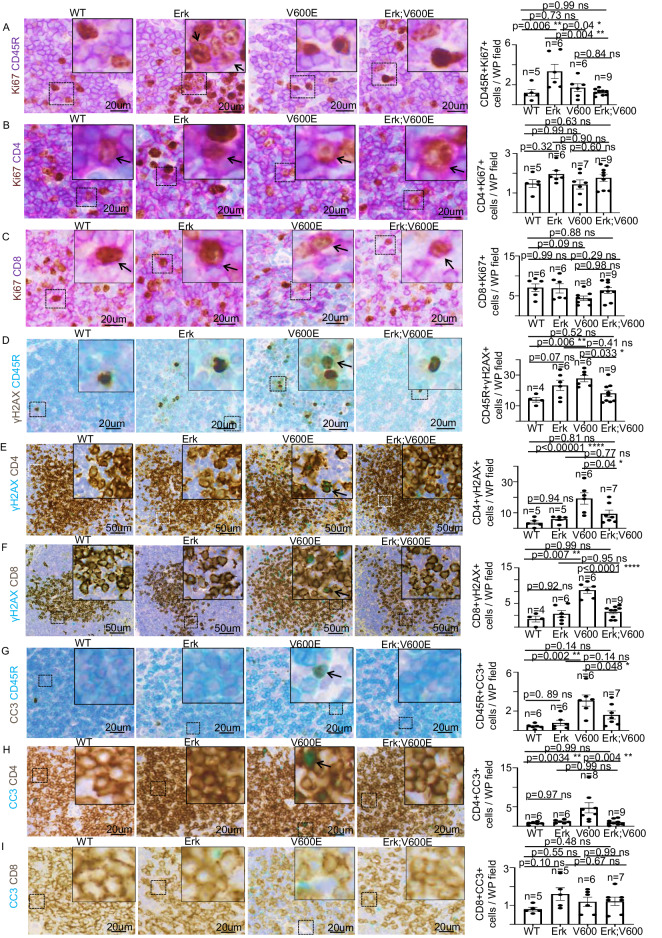
Genetic and molecular dissection of the role of ERK1/2 in BRAF^V600E^-dependent cell toxicity in splenic CD4+, CD8+, and CD45R+ lymphocytes. Representative images and quantifications showing double immunostaining aimed at detecting Ki67 in combination with either **A** CD45R, **B** CD4, or **C** CD8 markers in spleen sections of WT, Erk, BRAF^V600E^ and Erk;BRAF^V600E^ mice. Representative images and quantifications showing double immunostainings aimed at detecting γH2AX in combination with either **D** CD45R, **E** CD4, or **F** CD8 markers in spleen sections of WT, Erk, BRAF^V600E^, and Erk;BRAF^V600E^ mice. Representative images and quantifications showing double immunostaining aimed at detecting CC3 in combination with either **G** CD45R, **H** CD4, or **I** CD8 markers in spleen sections of WT, Erk, BRAF^V600E^ and Erk;BRAF^V600E^ mice. Quantifications were performed on at least five different areas of the sections in a random way. Data are expressed as mean ± SEM; *n* = animals per group. *P < 0.05; **P < 0.01; ***P < 0.001, ****P < 0.0001; ns not significant. (ANOVA test with Tukey’s post-hoc correction). Arrows point to selected positive cells for the indicated marker. Insets: magnifications of areas inside dashed squares.

To better understand how acute BRAF^V600E^ expression rapidly culminates in a drastic loss of spleen cellularity, we first asked whether such a cell toxicity might be mediated by ERK1/2 phosphorylation alteration. For this purpose, WB analysis from spleen total protein extracts revealed that in both Erk and Erk;BRAF^V600E^ mice, albeit ERK1 was not detectable, ERK2 protein levels were significantly reduced by 25–30% compared to WT strain (Fig. 3A). These data indicate that at the specific time-point analyzed (Fig. 2A), the Cre-dependent Erk2^lox/lox^ gene inactivation resulted in a partial, and yet statistically significant, reduction of ERK2 total protein levels (See Discussion). In line with this, immunohistochemistry experiments showed that total ERK1/2 protein is drastically reduced in the white pulp (WP) zone of Erk and Erk;BRAF^V600E^ mice compared to controls (Fig. 3B). Unexpectedly, even though BRAF^V600E^ activation resulted in no apparent change in ERK1/2 phosphorylation in either Erk;BRAF^V600E^ or BRAF^V600E^ mice compared to control strains (Fig. 3A), immunostaining experiments revealed that, upon BRAF^V600E^ activation, ERK1/2 phosphorylation was significantly increased in the red pulp (RP) and decreased in the WP zone (Supplementary Fig. 5A; Fig. 3C). Thus, these findings suggest that the BRAF^V600E^-induced cytotoxic response in splenic lymphocytes is associated to an overall reduction of ERK1/2 phosphorylation in the WP. Remarkably, constitutive Erk1 loss, coupled to 25–30% ERK2 protein level reduction, is sufficient to promote a drastic increase in Ki67+ WP cells, giving rise even to high density proliferative centers (Fig. 3D). However, such proliferation stimulation is completely rescued upon BRAF^V600E^ expression, even in Erk1^−/−^ background, thus suggesting that such proliferation alteration is not the main cause of BRAF^V600E^-triggered splenic lymphocyte depletion, and that upon BRAF^V600E^ activation, ERK1 becomes dispensable for the BRAF^V600E^-driven phenotype on lymphocytes proliferation (Fig. 3D). Conversely, no changes in p21^CIP1^ expression upon either ERK1 loss or BRAF^V600E^-activation were observed, thus ruling out the possibility that cell cycle arrest might play a role in BRAF^V600E^-dependent splenic lymphocyte depletion (Fig. 3E). Nevertheless, albeit BRAF^V600E^-expression results in robust DNA damage response (DDR) and apoptosis in the WP (Fig. 3F, G), the total and partial depletion of respectively ERK1 and ERK2 fully rescue BRAF^V600E^-dependent DDR and cell death induction, thus collectively suggesting that the immediate BRAF^V600E^-triggered splenic cell loss is not due to either proliferation inhibition, cell cycle arrest or cell death of resident splenic lymphocytes. Next, we set to identify the specific lymphocyte populations susceptible to the BRAF^V600E^-mediated cell response described in Fig. 3. For this purpose, an array of double immunostaining experiments by using the lymphocyte markers CD45R, CD4, and CD8 combined with Ki67, revealed that the proliferation cue elicited by ERK1/ERK2 depletion specifically encompasses B-, but not T-cells (Fig. 4A–C). However, BRAF^V600E^-activation, even in the Erk1^−/−^ background, fully rescues the proliferation increase observed in B-lymphocytes induced by loss of Erk1/2, thus indicating that residual ERK2 is sufficient to accomplish such a decrease in cell proliferation upon BRAF^V600E^ activation (Figs. 4A–C and 2D). Furthermore, additional double immunohistochemistry experiments with the above-mentioned lymphocyte markers in combination with γH2AX or CC3 also showed that, while BRAF^V600E^-activation induces an overall DDR activation in CD45R+, CD4+, and CD8+ cells, which is promptly rescued upon loss of ERK1/2 (Fig. 4D–F), only CD45R+ and CD4+, but not CD8+, lymphocytes show a significant increase of oncogene-induced cell death, which is reverted in an Erk1/2-dependent manner (Fig. 4G–I). Collectively, these findings strongly suggest that BRAF^V600E^-challenge effects over splenic lymphocytes are cell-specific and the impact of Erk1/2 loss in the BRAF^V600E^-triggered phenotypes is dependent on the specific biological function.

### Genetic and molecular dissection of ERK1/2 role in BRAF^V600E^-dependent cell toxicity in Club cells

**Fig. 5:**
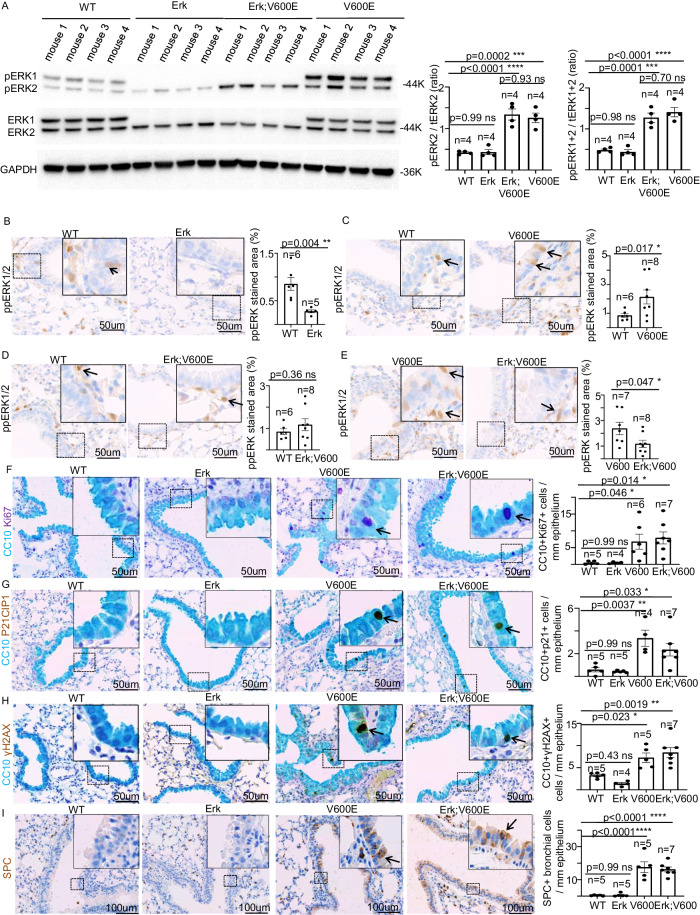
Genetic and molecular dissection of the role of ERK1/2 in BRAF^V600E^-dependent cell toxicity in Club cells. **A** Representative image (left) and quantifications (right) showing immunoblot experiments from lung protein extracts of WT, Erk, Erk;BRAF^V600E^ and BRAF^V600E^ mice. GAPDH is used as a loading control for the WB analysis. (ANOVA test with Tukey’s post-hoc correction). Representative images (left) and quantifications (right) of phosphorylated ERK1/2 immunostainings from lung sections of respectively **B** WT and Erk mice, **C** WT and BRAF^V600E^ mice, **D** WT and Erk;BRAF^V600E^ mice and **E** BRAF^V600E^ and Erk;BRAF^V600E^ mice. Quantifications of immunostainings were performed on at least five different areas of the sections in a random way. Data are expressed as mean ± SEM; *n* = animals per group. *P < 0.05; **P < 0.01; ***P < 0.001, ns = not significant. (T-Student’s test unpaired). Arrows point to selected positive cells for the indicated marker. Insets: magnifications of areas inside dashed squares. Representative images and quantifications showing double immunostainings aimed at detecting CC10 in combination with either **F** Ki67, **G** p21^CIP1^ or **H** γH2AX markers in lung sections of WT, Erk, BRAF^V600E,^ and Erk;BRAF^V600E^ mice. (**I**) Representative images and quantifications showing SPC immunostaining in lung sections of WT, Erk, BRAF^V600E^, and Erk;BRAF^V600E^ mice. Quantifications were performed on at least five different areas of the sections in a random way. Data are expressed as mean ± SEM; *n* = animals per group. *P < 0.05; **P < 0.01; ***P < 0.001, ****P < 0.0001; ns not significant. (ANOVA test with Tukey’s post-hoc correction). Arrows point to selected positive cells for the indicated marker. Insets: magnifications of areas inside dashed squares.

We recently provided evidence that acute BRAF^V600E^-expression induces cytotoxicity in several lung cellular types, including CCs [15]. To unravel the role of ERK1 and ERK2 phosphorylation in these BRAF^V600E^-induced phenotypes in the lung, we first performed both WB and immunohistochemical (IHC) analyses. These experiments revealed that in Erk and Erk;BRAF^V600E^ mice, albeit ERK1 was undetectable and ERK2 protein level is unaltered compared to WT strain, global ERK1/2 phosphorylation in the lung is significantly reduced only in Erk mice compared to WT strain (Fig. 5A–D; Supplementary Fig. 5B). In line with this, BRAF^V600E^ activation resulted in a dramatic increase in ERK1 and ERK2 phosphorylation, in both Erk;BRAF^V600E^ and BRAF^V600E^ mice compared to control strain (Fig. 5A, C, D). Importantly, BRAF^V600E^-dependent ERK2 phosphorylation increase in Erk;BRAF^V600E^ mice is comparable to that one observed in the presence of ERK1 (Fig. 5A, E), thus suggesting that ERK1 depletion is not counterbalanced by any relative over-compensation in ERK2 phosphorylation upon BRAF^V600E^-induction. These data also suggest that the solely phospho-ERK2 amount is sufficient to fully propagate downstream the BRAF^V600E^-triggered cytotoxic effect in the lung (see below). Next, to determine the role of ERK1/2 in BRAF^V600E^-induced CCs toxicity [15], we performed an array of immunostaining experiments by using the specific CC marker CC10 (CCs secretory protein 10 KDa) combined with either Ki67, P21^CIP1^ and γH2AX as well as the SPC ATII marker. These analyses confirmed that BRAF^V600E^-activation results in proliferation stimulation, DDR induction, cell cycle arrest, and CC-to-ATII transdifferentiation [15] (Fig. 5F–G). Remarkably, the constitutive Erk1 ablation is not capable of quenching the oncogenic-induced response, thus strongly suggesting that ERK1 is dispensable for the BRAF^V600E^-triggered cytotoxicity in CCs (Fig. 5F–G).

### Impact of combined genetic and chemical inhibition of ERK1/2 on BRAF^V600E^-induced phenotypes in the lung

**Fig. 6:**
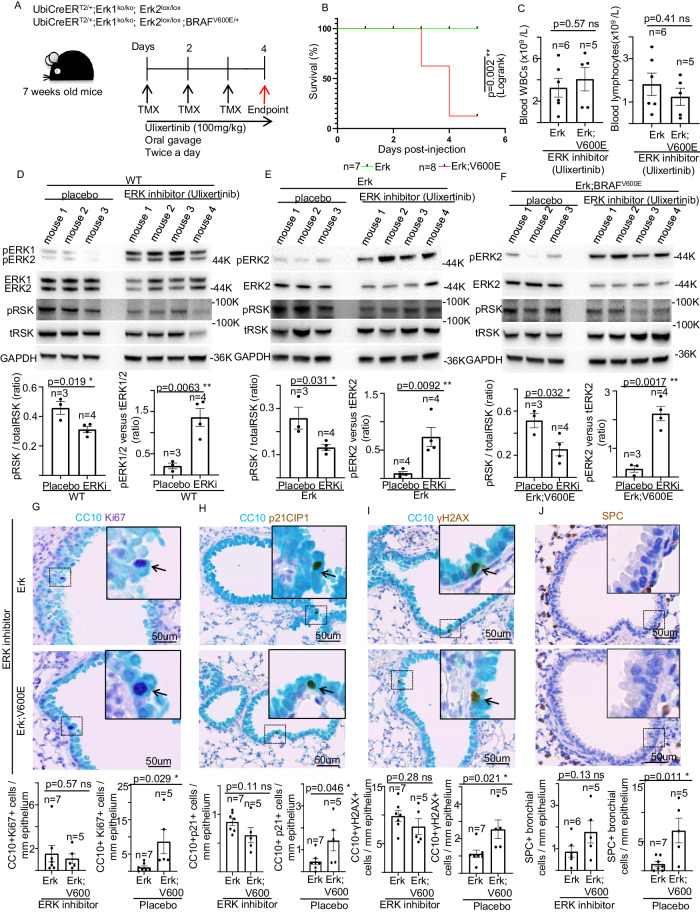
Impact of combined genetic and chemical inhibition of ERK1/2 on BRAF^V600E^-induced phenotypes in the lung and Club cells. **A** Schematic representation of mouse genotypes and experimental procedure employed in this study. TMX = 4-hydroxy tamoxifen. **B** Survival curves of mice with the indicated genotype. n= animals per group. **P < 0.001. (Log Rank test). **C** Charts showing the number of total circulating white blood cells (left) and lymphocytes (right) of the indicated experimental groups upon TMX and ulixertinib treatment. Representative images (top) and quantifications (bottom) showing immunoblot experiments from lung protein extracts of respectively TMX-administered **D** WT, **E** Erk, **F** Erk;BRAF^V600E^ mice treated with either ulixertinib or placebo. GAPDH is used as a loading control. ERKi ERK inhibitor (ulixertinib). tERK total ERK, ppERK phosphorylated ERK, tRSK total RSK, pRSK phosphorylated RSK. Data are expressed as mean ± SEM; *n* = animals per group. *P < 0.05; **P < 0.01; ***P < 0.001, ns not significant. (T-Student’s test unpaired). **G**–**J** Representative images and quantifications showing double immunostainings aimed at detecting CC10 in combination with either **G** Ki67, **H** p21^CIP1^, or **I** γH2AX markers in lung sections of TMX-administered Erk and Erk;BRAF^V600E^ mice treated with either ulixertinib or placebo. Note that the corresponding representative images for the placebo treatments are shown in Supplementary Fig. 7A–C. **J** Representative images and quantifications showing SPC immunostaining in lung sections of TMX-administered Erk and Erk;BRAF^V600E^ mice treated with either ulixertinib or placebo. Note that the corresponding representative images for the placebo treatments are shown in Supplementary Fig. 7D. Quantifications were performed on at least five different areas of the sections in a random way. Data are expressed as mean ± SEM; *n* = animals per group. ns not significant. (T-Student’s test unpaired). Arrows point to selected positive cells for the indicated marker. Insets: magnifications of areas inside dashed squares.

**Fig. 7:**
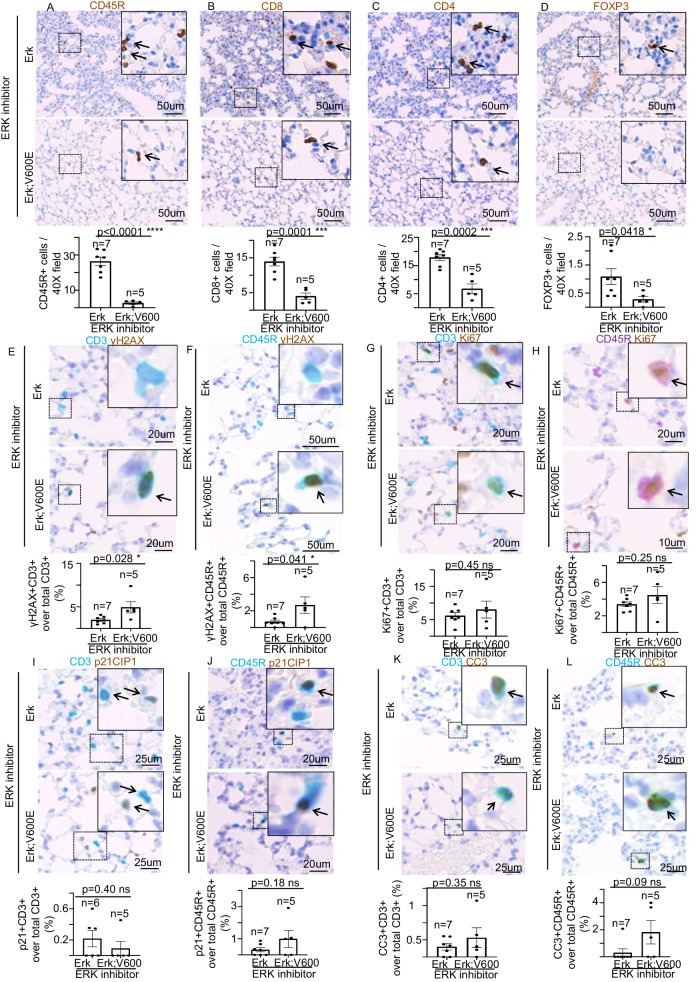
Impact of combined genetic and chemical inhibition of ERK1/2 on BRAF^V600E^-induced phenotypes in lung lymphocytes. Representative images and quantifications showing **A** CD45R, **B** CD8, **C** CD4, and **D** FOXP3 immunostainings in lung sections of TMX-administered Erk and Erk;BRAF^V600E^ mice treated with ulixertinib. Representative images and quantifications showing double immunostainings aimed at detecting **E**, **F** γH2AX, **G**, **H** Ki67, **I**, **J** p21^CIP1^ and **K**, **L** CC3 in combination with either (**E**, **G**, **I**, **K**) CD3 or (**F**, **H**, **J**, **L**) CD45R markers in lung sections of TMX-administered Erk, and Erk;BRAF^V600E^ mice treated with ulixertinib. Quantifications were performed on at least five different areas of the sections in a random way. Data are expressed as mean ± SEM; *n* = animals per group. *P < 0.05; **P < 0.01; ***P < 0.001, ****P < 0.0001; ns not significant. (T-Student’s test unpaired). Arrows point to selected positive cells for the indicated marker. Insets: magnifications of areas inside dashed squares.

At their end-point TMX-treated Erk, BRAF^V600E^ mice display mild or no alterations in ERK2 total protein levels (Figs. 3A and5A, C). Thus, in order to accomplish a significant inhibition of ERK2 activity, which would allow us to address the impact of the complete inhibition of both ERK1/ERK2 on BRAF^V600E^-dependent responses, we administered TMX-treated Erk;BRAF^V600E^ mice the selective ERK1/2 inhibitor ulixertinib (BVD-523; VRT752271) (Fig. 6A). Intriguingly, albeit even a combined ERK1/2 genetic and chemical inhibition failed to ameliorate the acute BRAF^V600E^-induced lethality (Fig. 6B), ulixertinib, but not placebo, treatment fully rescued the BRAF^V600E^-dependent loss of blood lymphocytes (Fig. 6C, Supplementary Fig. 6A, B), without affecting other WBCs (Supplementary Fig. 6C–J). These findings suggest that at such a dosage, ERK2 chemical inhibition can only partially inhibit the dramatic effects of the BRAF^V600E^-mediated ubiquitously activated ERK-signaling. However, WB analysis from lung protein extracts of WT, Erk and Erk;BRAF^V600E^ mice revealed that upon ulixertinib treatment, whereas phosphorylated fraction of the ERK target RSK is significantly decreased, ERK1/2 phosphorylated fractions are paradoxically increased concomitantly to a significant reduction of total protein ERK1/2 levels compared to the placebo-treated groups, which are three known consequences of ulixertinib-mediated ERK inhibition [29–34] (Fig. 6D–F, Supplementary Fig. 6K–M). These data indicate that, at least in the lung, ERK2 chemical inhibition is efficiently accomplished. As a consequence of that, BRAF^V600E^-triggered toxicity in CCs, such as proliferation stimulation, DDR activation, apoptosis induction, and CC-to-SPC transdifferentiation, is completely abrogated in TMX-treated Erk, BRAF^V600E^ mice upon ulixertinib, but not placebo, administration, therefore strongly suggesting that ERK2 is sufficient to propagate the aberrant BRAF^V600E^-signaling resulting in CCs toxicity (Fig. 6G–J, Supplementary Fig. 7A–D). Surprisingly, as shown in Fig. 7A–D and Supplementary Fig. 7E–H, the overall loss of pulmonary lymphocytes observed in BRAF^V600E^ and Erk;BRAF^V600E^ mice is not rescued even following treatment with either ulixertinib or the corresponding placebo, therefore indicating that the combined ERK1/2 genetic and chemical inhibition is not sufficient to restore the physiological levels of lung lymphocytes. Interestingly, additional double immunostaining experiments of either CD3 or CD45R markers combined with γH2AX also revealed that in the absence of ERK-chemical inhibition, lymphocytes from Erk;BRAF^V600E^ strain display robust DDR activation compared to the corresponding cells from Erk mice. These findings suggest that, similarly to CCs, ERK1 is dispensable for the BRAF^V600E^-mediated γH2AX enrichment for lung lymphocytes as well (Supplementary Fig. 8A, B). However, contrary to what observed in CCs, even upon ulixertinib/placebo treatments, pulmonary parenchyma as well as both lung B- and T-lymphocytes from Erk;BRAF^V600E^ mice still showed significantly higher levels of DNA damage compared to Erk strain (Supplementary Fig. 9A–D, Fig. 7E, F), therefore indicating that the ability of ERK1/2 chemical inhibition to prevent BRAF^V600E^ from yielding DDR activation is cell-specific. Intriguingly, albeit both the ulixertinib- and the placebo-treated Erk;BRAF^V600E^ mice showed increased levels of p21^CIP1^, but not of CC3, in the lung parenchyma compared to Erk mice (Supplementary Fig. 9E–H), no induction of either cell cycle arrest or apoptosis was observed upon BRAF^V600E^ expression in pulmonary B- and T-lymphocytes (Fig. 7G–L, Supplementary Fig. 10A–F). Such findings suggest that the non-rescuable BRAF^V600E^-dependent lymphocyte depletion is not ascribable to any alteration in either cell cycle progression or apoptosis of resident lung lymphocytes.

## Discussion

**Fig. 8:**
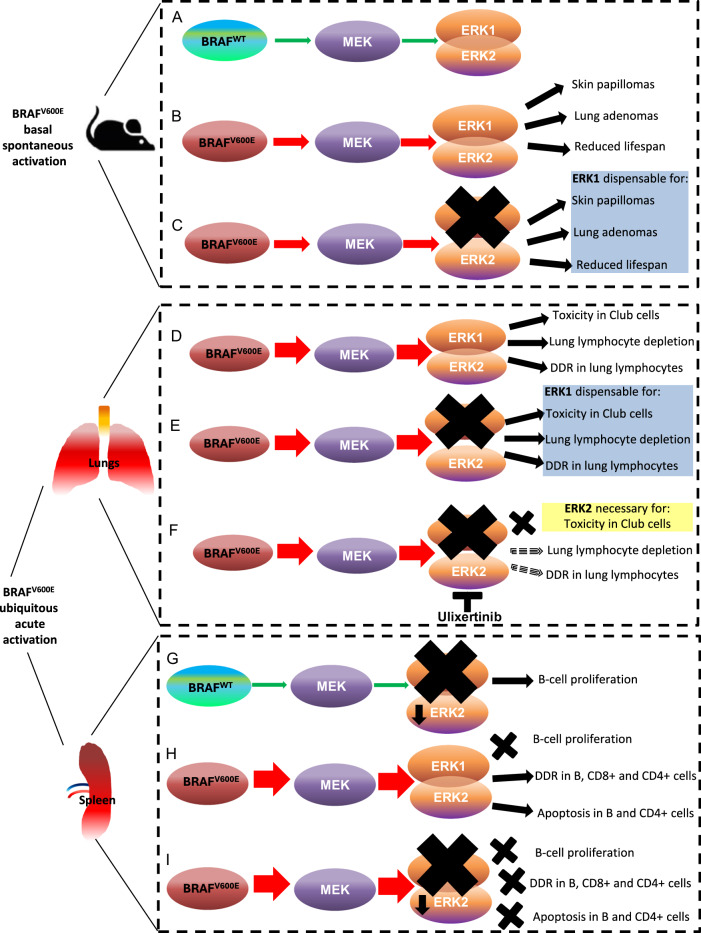
Schematic representation of the differential roles of ERK1/ERK2 kinases in BRAF^V600E^-triggered phenotypes in adult mouse models. **A**–**C** Schematic diagram showing how ERK1/ERK2 kinases contribute to the BRAF^V600E^-triggered phenotypes that are dependent on the spontaneous basal activation of BRAF^V600E^ allele in TMX-untreated adult mouse models. **A** ERK-pathway signaling in physiological conditions. **B** BRAF^V600E^ promotes ERK-pathway hyperactivation which results in the development of spontaneous skin papillomas and lung adenomas as well as reduced lifespan. **C** Constitutive loss of ERK1 does not affect the BRAF^V600E^-triggered phenotypes described in (**B**). **D**–**F** Schematic diagram showing how ERK1/ERK2 kinases contribute to the BRAF^V600E^-triggered phenotypes in the lung that are observed upon acute ubiquitous activation of oncogenic BRAF. **D** BRAF^V600E^ expression results in Club cells toxicity, lung lymphocytes depletion, and DNA damage response (DDR) activation in the lung lymphocytes. **E** Constitutive loss of ERK1 does not affect the BRAF^V600E^-triggered phenotypes described in (**D**). **F** Constitutive loss of ERK1, coupled to ERK2 chemical inhibition, rescues the BRAF^V600E^-triggered phenotypes in Club cells, but not either the depletion or DDR activation in pulmonary lymphocytes. **G**–**I** Schematic representation of how ERK1/ERK2 kinases contribute to the BRAF^V600E^-triggered phenotypes in the spleen that are observed upon acute ubiquitous BRAF^V600E^ activation. **G** Constitutive loss of ERK1, coupled to 25–30% reduction in ERK2 expression, results in splenic B-lymphocyte proliferation. **H** BRAF^V600E^ activation results in the ERK1-dependent proliferation increase of B cells, DDR activation in T- and B-lymphocytes as well as apoptosis induction in B- and T-helper cells. **I** Constitutive loss of ERK1, coupled to 25–30% reduction in ERK2 expression, rescues the BRAF^V600E^-triggered phenotypes described in (**H**). (DDR DNA damage response).

In this report we uncovered the selective contribution of ERK1/ERK2 kinases in multiple BRAF^V600E^-driven responses in vivo (Fig. 8). We unveiled that, albeit ERK1 is dispensable for both BRAF^V600E^-induced lethality and the growth of BRAF^V600E^-driven tumors, it might play a role in lung adenoma initiation. Similarly to ERK1, in a skin tumor mouse model, it has been shown that MEK1 deletion partially inhibits papilloma development [35]. However, we do not rule out the possibility that a random activation of BRAF^V600E^ might concomitantly occur occasionally with abrogation of ERK2. Consequently, such a hypothetical ERK2 inactivation might impair the early stages of adenoma development and result in a reduced number of lung tumors in the Erk;BRAF^V600E^ mice.

Our evidence indicating that global phosphorylated ERK2 levels in the spleen remain unchanged in Erk;BRAF^V600E^ and BRAF^V600E^ models is very likely the result of the opposite effects that BRAF^V600E^ expression has over ERK phosphorylation in the different splenic zones. More specifically, whereas BRAF^V600E^ leads to an increase of phosphorylated ERK2 in the red pulp, it yields a significant reduction of ERK2 phosphorylation in the WP. Furthermore, we found that BRAF^V600E^ activation rapidly results in splenic lymphocyte apoptosis. Thus, it is arguable that the reduction in ERK phosphorylation in WP following BRAF^V600E^ activation might be ascribable to the dramatic loss of cellularity due to the oncogenic-dependent apoptosis.

We also unveiled that ERK1/ERK2 have different impacts on BRAF^V600E^-elicited phenotypes in lymphocytes. In fact, ERK1 systemic ablation, coupled to partial (~30%) ERK2 loss, robustly and specifically stimulates splenic B-lymphocyte proliferation only in the absence of BRAF^V600E^-activation. On the other hand, ERK1/2 loss is sufficient to prevent BRAF^V600E^-challenged cells from undergoing DDR activation and apoptosis in the WP zone (Fig. 8). These findings let us envisage that the ERK1/ERK2 contribution to the BRAF^V600E^-driven acute phenotypes in vivo is dynamic and varies on the basis of the cell type (see below), the specific biological function and the level of ERK-pathway activation.

The epistasis of BRAF^V600E^-activation over ERK2 depletion in several acute phenotypes is likely due to the fact that the time window compatible with mice viability is not sufficient to promote an efficient ERK2 deletion. Consequently, as ERK2 is an extremely stable protein [36], BRAF^V600E^-activation may occur more rapidly than the ability of mouse cells to degrade the residual ERK2 molecules produced before Cre-mediated Erk2 gene disruption. Hence, in this time window also in Erk;BRAF^V600E^ mice, BRAF^V600E^ may ensure an efficient cue propagation downstream. In line with this notion, our experiments in the spleen show that 6 days post-TMX injections, whereas BRAF^V600E^ allele has been efficiently activated, Cre-dependent Erk2^lox/lox^ gene inactivation only resulted in a ~25% reduction of ERK2 total protein levels.

When expressed in the hematopoietic progenitors by using the interferon-inducible Mx-promoter-driven Cre, BRAF^V600E^ provokes splenomegaly [7, 37] and hairy cell leukemia-like disease in mice [37]. However, this blood cancer phenotype becomes apparent only several weeks after BRAF^V600E^-activation and induces no decrease in lymphocyte number [37]. Thus, it is arguable that the rapid BRAF^V600E^-triggered lymphocyte depletion in our model might be ascribable to the BRAF^V600E^-provoked alteration of some intermediate phase(s) involving lymphocyte development or to the effects of BRAF^V600E^-expression in some cell type(s) necessary for lymphocyte homeostasis (see below). Moreover, the discrepancy observed in the BRAF^V600E^-dependent phenotypes in lymphocytes between our model and those ones described [7, 37] might depend on the different promoters employed to drive Cre-expression as well as on the method for Cre induction. Indeed, a ubiquitin promoter-driven Cre activation might result in BRAF^V600E^-expression also in cellular types that are unresponsive to Mx-promoter-driven [7] Cre basal activation. Furthermore, an acute TMX-mediated Cre activation would also result in a more rapid and vigorous overall BRAF^V600E^-activation, which might give rise to such previously uncharacterized phenotypes.

Although upon antigenic overstimulation, ERK-pathway activation promotes cell cycle arrest in lymphocytes in vitro by eliciting p21^CIP1^ expression [38, 39] in the absence of apoptosis induction [38], the ERK1/ERK2 contribution in BRAF^V600E^-mediated ERK-pathway aberrant activation in lymphocytes has yet to be elucidated. Here we disclosed that ubiquitous BRAF^V600E^ acute activation provokes an immediate overall depletion of blood, splenic, and lung lymphocytes. Such a lymphocyte loss occurs independently of ERK1, does not rely on either p21^CIP1^-induced cell cycle arrest or CC3-mediated apoptosis, and is partially unresponsive to ERK1/2 chemical inhibition.

Importantly, BRAF^V600E^ lesion is known to cause 100% of the cases of B-cell hairy cell leukemia [1, 40, 41] and has been found in other B- or T-cell malignancies such as chronic lymphocytic [42] and prolymphocytic [42] leukemias, non-Hodgkin’s lymphomas [43], diffuse large B cell lymphoma [44] and multiple myeloma [44]. Thus, altogether, our findings provide useful insights into the comprehension of the pathophysiology of a plethora BRAF^V600E^-driven malignancies [1–4, 45], including the above-mentioned blood cancers. Additionally, the use of Erk;BRAF^V600E^ model in combination with tissue-specific promoters will be a valuable tool in vivo to genetically dissect the ERK1/2 selective contribution in cancer development in a variety of BRAF^V600E^-driven tumor murine models [7, 8, 10–14].

Ulixertinib is currently being tested in clinical trials [46], and its use for the treatment of mutant BRAF-triggered malignancies is promising [32, 47]. However, to date, the impact of ulixertinib on systemic BRAF^V600E^-triggered acute phenotypes in vivo is unknown. We showed for the first time in vivo that, albeit ERK1 is dispensable for the BRAF^V600E^-triggered cytotoxicity in CCs [15], ERK2 chemical inhibition fully abrogates these detrimental effects in bronchial cells (Fig. 8). Conversely, we also revealed that ulixertinib does not attenuate BRAF^V600E^-mediated DDR activation in lung lymphocytes even in the absence of ERK1. These findings may indicate that the impact of the genetic and chemical ERK1/2 inhibition on BRAF^V600E^-triggered DNA damage in vivo is dictated by the sensitivity of a specific cellular type to ERK-pathway abrogation. Alternatively, the unresponsiveness to the ERK inhibition of BRAF^V600E^-elicited DDR activation in lymphocytes might also underlie the existence of a potential ERK1/2-independent mechanism for BRAF^V600E^ in the DDR induction in vivo. In support of this, recent work showed an ERK1/2-independent role for BRAF in the DDR [48, 49] in vitro.

The evidence that ulixertinib does not affect either the early lethality or lung lymphocyte depletion upon BRAF^V600E^-activation let us hypothesize that the drug posology used for the treatment may not be sufficient to rescue some acute BRAF^V600E^-dependent phenotypes. Thus, it is conceivable that such level of ERK-inhibition might not be capable of restoring some BRAF^V600E-^challenged upstream cellular processes for which ERK-pathway physiologically plays a crucial role, such as lymphocyte activation [50], survival [51], development [52, 53], differentiation [54, 55] or maturation [18, 56] which would culminate in lymphocyte loss. Further studies will be required to identify which lymphocyte maturation step is most affected by BRAF^V600E^ activation.

Collectively, our data let us envisage the existence of unexplored efficacy limitations as well as previously uncharacterized heterogeneous outcomes to ulixertinib treatment due, at least in part, to the diversity of cell-specific responses to ERK-chemical inhibition in vivo. Hence, our findings will provide helpful insights into the understanding of the consequences in vivo and the improvement of the novel ERK-pathway targeted anti-cancer therapies.

## Materials and methods

### Murine models

BRAF^LSLV600E^, Erk1^−/−^, and Erk2^lox/lox^ mice were described previously [3, 6, 7, 17, 18, 57]. These mouse models were crossed with a mouse strain carrying ubiquitously expressed, TMX-activated recombinase, UBC-CreER^T2^ [16], to generate UBC-CreER^T2/+^, Erk2^lox/lox^, Erk1^−/−^; UBC-CreER^T2/+^;BRAF^LSL_V600E/+^ and UBC-CreER^T2/+^;Erk2^lox/lox^; Erk1−/−; BRAF^LSL_V600E/+^ mice. All mice were maintained at the Spanish National Cancer Research Center under specific pathogen-free conditions in accordance with the recommendations of the Federation of European Laboratory Animal Science Associations (FELASA). All animal experiments were approved by our Institutional Animal Care and Use Committee (IACUC) and by the Ethical Committee for animal experimentation (CEIyBA) (PROEX 106.7/20). We followed the Animal Research Reporting of in Vivo Experiments (A.R.R.I.V.E.) guidelines developed by the National Center for the Replacement, Refinement & Reduction of Animals in Research (NC3Rs). Both male and female mice, with mixed backgrounds, were used for the experiments.

### Mouse treatments

The mice received intraperitoneal injections of 4-hydroxy TMX (Sigma H6278) (1 mg/injection, 3 injections, 1 injection per day for 3 consecutive days).

Ulixertinib (VRT752271, HY-15816, MedChemExpress) was resuspended at the concentration of 10 mg/mL in 5%DMSO, 40%PEG300, 5% Tween80 and 50% saline solution according to the manufacturer’s instructions and was administered 100 mg/kg by oral gavage twice a day (200 mg/kg/day), as previously described [58].

The phenotypical analysis of the TMX-administered mice treated with either placebo or ulixertinib was performed a day before (endpoint = day 4) compared to the experiments carried out in the mice treated only with TMX (endpoint = day 5, Figs. 2–5). The reason of this choice has been dictated by the observation that the Erk;BRAF^V600E^ and BRAF^V600E^ mice treated with both TMX and Placebo/ERKi display a shorter survival (Fig. 6B) and consequently, at Day 5 post treatment the vast majority of them were dead. By choosing Day 4 as an endpoint, we reached an ethically justifiable compromise which complies with FELASA and ARRIVE guidelines to limit animal suffering and simultaneously to allow the generation of samples scientifically suitable for the downstream phenotypical analyses.

### Peripheral blood analyses

Blood was collected by intracardiac puncture using EDTA-containing tubes (Aquisel). Automated peripheral blood counts were obtained using an Auto Hematology Analyzer Model LaserCell (CVM) according to standard manufacturer’s instruction.

### Immunohistochemistry analyses in tissue sections

Tissues were fixed in 10% buffered formalin, embedded in paraffin wax, and sectioned at 5 mm. For histological examination, sections were stained with hematoxylin and eosin, according to standard procedures as previously described [59–62]. CC3 Asp175 (Cell Signaling Technology 9661), CC10 (Santa Cruz Biotechnology sc-9772), CD4 [15, 60, 63] (1:50, Clone D7D2Z, 25229, Cell Signaling Technology), CD8 [60, 62] (1:200, Clone 94A, CNIO Monoclonal Antibodies Core Unit handmade), prosurfactant protein C (millipore AB3786), p21 (291 H/B5, CNIO Monoclonal antibodies facility homemade), γH2AX Ser 139 (Millipore 05-636), PPERK Thr202/Tyr204 (Cell Signaling Tehcnology 9101), Ki67 (Cell Signaling 12202), total ERK1/2 (Abcam ab54230, ERK-7D8), F4-80 (ABD Serotec MCA497), CD3 [64] (undiluted, Roche, 2GV6, ref.790–4341); CD45R/B220 [65] (1:150, BD biosciences, 557390, RA3-6B2); FOXP3 [62, 66] (1:50, 221D, CNIO Monoclonal antibodies facility homemade), p53 (POE316A, CNIO Monoclonal antibodies facility homemade), antibodies were used for immunohistochemistry in tissue sections. The antibodies used to identify the different lymphocytes subpopulations were previously tested, validated, and used by us [15, 60, 62] after the proper testing and validation performed by the corresponding manufacturers [63–66]. Pictures were taken using Olympus AX70 microscope. The percentage of positive cells was identified by eye and the areas were calculated by ImageJ and Zen 3.1 (Zeiss) softwares.

### Protein extract preparation and western blot

Protein extracts were obtained as follows: 45 mg of lung for each mouse (10 mg for papillomas) were mechanically homogenized in 20 µl/mg of lysis buffer (50 mM TrisHCl pH 7.5, 420 mM NaCl, 1% Triton, 1 mM EDTA, 2.5 mM MgCl2, cOmplete protease inhibitor cocktail (Roche), Pierce phosphatase inhibitor mini tablets (ThermoScientific), protease and phosphatase inhibitor cocktail 100X (Sigma), in *BERTIN Precellys 24 Lysis & Homogenization* machine, incubated 30 min on ice in agitation, sonicated 10 s, centrifuged at 14,000× *g* for 20 min at 4 °C. The recovered supernatant was passed through a 0.22 filter, aliquoted, flash-frozen in liquid nitrogen, and stored at –80 °C. Protein concentration was determined using the Bio‐Rad DC Protein Assay (Bio‐Rad). 40 µg of nuclear protein extracts were separated in SDS–-polyacrylamide gels by electrophoresis. After protein transfer onto nitrocellulose membrane, the membranes were blocked with 5% non-fat dried milk or 5% BSA (for the detection of phosphorylated proteins) both resuspended in TBS-Tween 0.2% (TBT) and then incubated with the indicated antibodies diluted in TBT: monoclonal anti-actin 1:5000 (A5441, Sigma), anti-phospho Thr202/Tyr204 p44/42MAPK (ERK1/2) 1:1000 (Cell Signaling Technology 9101), anti-total ERK1 1:2500 (BD Pharmingen; 554100), anti-total ERK2 1:5000 (BD biosciences, 610103), anti-phospho p90RSK (pRSK) (Thr359/Ser363) 1:1000 (Cell Signaling, 9344), anti-RSK1/2/3 (total RSK) 1:1000 (Cell Signaling, 9355). Anti-phospho RSK and total RSK antibodies were incubated in 5%BSA in TBS-0.1%Tween and the corresponding WBs were washed with TBS-0.1% Tween. Antibody binding was detected after incubation with a secondary antibody coupled to horseradish peroxidase using chemiluminescence with ECL detection KIT (GE Healthcare) with Chemidoc (Biorad). For the quantification, protein‐band intensities were quantified by densitometric analysis with ImageLab software (Biorad). The total levels of each protein analyzed have been normalized versus actin and the mean of the specific protein/actin ratio deriving from at least 3 different replicates has been used to generate the chart as previously described [62, 67, 68].

### PCR

DNA of tissue samples was extracted using Phenol:Chloroform:Isoamyl:Alcohol (Sigma). We determined Cre-mediated recombination by using the following PCR program: 94 °C for 3 min, followed by 33 cycles of 94 °C denaturation for 25 s, 25 s annealing at 55 °C, elongation at 73 °C for 45 s, followed by a 4 min 73 °C elongation step with the following primes: Fw 5′-TGAGTATTTTTGTGGCAACTGC and Rev 5′-CTCTGCTGGGAAAGCGGC. This oligonucleotide primer pair hybridizes in intron 14 flanking the cassette insertion site. These conditions produce diagnostic PCR products of 185 bp for the wild-type BRAF and 308 bp for BRAF^V600E^alleles and a 335 bp PCR product for the Cre-activated BRAF^V600E^ allele. The samples were resolved in a 3% agarose gel and detected with GelDoc (BioRad) as previously described [69].

### Quantification and statistical analysis

Immunohistochemistry quantifications were performed by direct cell counting by using Zen3.1 Zeiss, QPath 2.0 and Image J softwares Unpaired Student’s *t*-test (two-tailed), ANOVA followed by Tukey’s post-hoc correction, Log Rank test were used to determine statistical significance. *P* values of less than 0.05 were considered significant. *p < 0.05, **p < 0.01, ***p < 0.001, ****p < 0.0001. Statistical analysis was performed using Microsoft® Excel 2016 and GraphPad/PRISM8 as previously described [70]. For animal studies no blinding/randomization was done/used. The number of mice per each experiment as well as the size of the experiments were obtained by performing power analysis.

### Supplementary information

Supplementary Figure Legends

Supplementary Figure 1

Supplementary Figure 2

Supplementary Figure 3

Supplementary Figure 4

Supplementary Figure 5

Supplementary Figure 6

Supplementary Figure 7

Supplementary Figure 8

Supplementary Figure 9

Supplementary Figure 10

Uncropped WBs

## Data Availability

The datasets and other information that support the findings of this study are available from the corresponding author upon reasonable request. Raw western blots are available in the Supplemental file.
