# Supplementary material for: Differential contribution for ERK1 and ERK2 kinases in BRAFV600E-triggered phenotypes in adult mouse models
Source: Cell Death Differ. 2024 May 2;31(6):804–19. doi: 10.1038/s41418-024-01300-x (PMC11165013; doi:10.1038/s41418-024-01300-x)
Supplement: Supplementary file 1 — Supplementary Figure Legends [file 41418_2024_1300_MOESM1_ESM.docx]

**Differential contribution for ERK1 and ERK2 kinases in BRAF^V600E^-triggered phenotypes in adult mouse models**

Giuseppe Bosso^1^, Ana Carolina Cintra Herpst^1^, Oscar Laguía^1^, Sarah Adetchessi^1^, Rosa Serrano^1^ and Maria A. Blasco^1*^

**Short title: Differential impact for ERK1/ERK2 in the BRAF^V600E^-driven responses *in vivo***

^1^ Telomeres and Telomerase Group, Molecular Oncology Program, Spanish National Cancer Centre (CNIO), Melchor Fernández Almagro 3, Madrid, E-28029, Spain.

* *Correspondence:* Maria A. Blasco

Spanish National Cancer Research Centre (CNIO)

3 Melchor Fernandez Almagro

Madrid E-28029, Spain

Tel.: +34.91.732.8031

Fax: +34.91.732.8028

Email: [mblasco@cnio.es](mailto:mblasco@cnio.es)

**Supplementary Figure 1. Impact of genetic inhibition of ERK1 on spontaneous BRAF^V600E^-induced tumors.**

(**A**) Representative image of TMX-untreated WT and Erk mice. (**B**) PCR analysis from papillomas derived from BRAF^V600E^ and Erk;BRAF^V600E^ mice in the absence of tamoxifen administration to detect spontaneous rearrangement of BRAF^LSL_V600E^ allele. Recombination of the BRAF^V600E^ allele was detected in papilloma-like skin lesion of untreated 11 weeks old BRAF^V600E^ mice but not in the brain which has been used as a negative control. (**C**) Representative images showing low and high magnifications of H/E stainings of lung sections of TMX-untreated WT, Erk, BRAF^V600E^ and Erk;BRAF^V600E^ mice. The arrows point to some lung adenomas. (**D**) Representative images and quantifications showing immunoblot experiments from lung protein extracts of TMX-untreated WT, Erk, BRAF^V600E^, Erk;BRAF^V600E^ mice. GAPDH is used as a loading control. pERK=phosphorylated ERK. A.U.=arbitrary units. Data are expressed as mean ± SEM; n= animals per group. *P<0.05; ** P<0.01; *** P<0.001, ns= not significant. (T-Student’s test unpaired for total and phosphor-ERK1 analysss, ANOVA test with Tukey’s post-hoc correction for total and phospho-ERK2 analyses).

**Supplementary Figure 2. Erk1 systemic genetic abrogation does not prevent spontaneous BRAF^V600E^ activation to give rise to lung adenomas.**

(**A**) Representative images and quantifications showing total ERK1/2 immunostaining in lung sections of TMX-untreated WT, Erk, BRAF^V600E^ and Erk; BRAF^V600E^ mice. (**B-D**) Representative images and quantifications showing (**B**) Ki67, (**C**) p21CIP1 and (**D**) CC3 immunostainings in lung sections of TMX-untreated BRAF^V600E^ and Erk; BRAF^V600E^ mice. Quantifications were performed on at least five different areas of the sections in a random way. Data are expressed as mean ± SEM; n= animals per group. * P<0.05; ** P<0.01; *** P<0.001, **** P<0.0001; ns= not significant. ANOVA test with Tukey’s post-hoc correction was used for (**A**) and T-Student’s test unpaired for (**B-D**). Arrows in (**A**) point to lung adenomas. Dashed circles delimit adenoma area in (**A**). Arrows in (**B-D**) point to selected positive cells for the indicated marker. Insets: magnifications of areas inside dashed squares.

**Supplementary Figure 3. Acute BRAF^V600E^ expression *in vivo* is epistatic over ERK1.**

(**A**) Survival curves of mice with the indicated genotype. n= animals per group. **** P<0.0001; ns= not significant (Log Rank test). TMX= 4-hydroxy tamoxifen. (**B**) Chart representing the ratio of the weights of the mice with the indicated genotypes at the day 1 and day 4 of the TMX treatment. (**C**) PCR analysis from lung and spleen derived from BRAF^V600E^, Erk;BRAF^V600E^ and WT mice to detect rearrangement of BRAF^LSL_V600E^ allele upon TMX administration. Recombination of the BRAF^V600E^ allele was detected in all the organs analyzed. (**D**) Charts showing the number of blood neutrophils, monocytes, eosinophils and basophils (from the left to the right) and of the indicated experimental groups upon TMX treatment. (**E**) Quantification of CD3 immunostaining shown in Figure 2D in spleen sections from TMX-treated WT, Erk, BRAF^V600E^ and Erk; BRAF^V600E^ mice. Data are expressed as mean ± SEM; n= animals per group. * P<0.05; ** P<0.01; *** P<0.001, **** P<0.0001; ns= not significant. (ANOVA test with Tukey’s post-hoc correction).

**Supplementary Figure 4. ERK1 abrogation in the presence of basal BRAF^V600E^ expression does not affect lymphocyte occurrence in the lung parenchyma.**

(**A-B**) Representative images and quantifications showing (**A**) CD45R and (**B**) CD3 immunostainings in lung sections of TMX-untreated WT and BRAF^V600E^ mice. Quantifications were performed on at least five different areas of the sections in a random way. Data are expressed as mean ± SEM; n= animals per group. ns= not significant. (T-Student’s test unpaired). Arrows point to selected positive cells for the indicated marker. Insets: magnifications of areas inside dashed squares.

**Supplementary Figure 5. BRAF^V600E^ activation in the spleen specifically elicit ERK phosphorylation in the red pulp zone and TMX-treatment at BRAF^V600E^ and Erk;BRAF^V600E^ mice endpoint does not result in significant decrease of ERK2 protein levels in the lung.**

(**A**) Representative images and quantifications showing phosphorylated ERK1/2 immunostainings in spleen sections of WT and BRAF^V600E^ mice. Quantifications were performed on at least five different areas of the sections in a random way. Data are expressed as mean ± SEM; n= animals per group. * P<0.05 (T-Student’s test unpaired). Arrows point to selected positive cells for the indicated marker. Insets: magnifications of areas inside dashed squares. The dashed lines mark the boundary between white and red pulps.(**B**) Quantifications of immunoblot experiment shown in Figure 5A from lung protein extracts of TMX-administered WT, Erk, Erk;BRAF^V600E^ and BRAF^V600E^ mice. Data are expressed as mean ± SEM; n= animals per group. ns= not significant. (ANOVA test with Tukey’s post-hoc correction).

**Supplementary Figure 6. Effects of ERK1 abrogation and ulixertinib/placebo treatments on blood leukocytes and lung parenchyma**

(**A-F**) Charts showing the number of (**A**) circulating white blood cells, (**B**) blood lymphocytes, (**C**) neutrophils, (**D**) monocytes, (**E**) eosinophils and (**F**) basophils (from the left to the right) and of the indicated experimental groups upon TMX and placebo treatments. (**G-J**) Charts showing the number of (**G**) blood neutrophils, (**H**) monocytes, (**I**) eosinophils and (**J**) basophils (from the left to the right) and of the indicated experimental groups upon TMX and ulixertinib treatments. (**K-M**) Charts showing the quantifications of GAPDH-normalized total ERK1/2 levels of the western blot experiments shown in Figure 6D-F. Data are expressed as mean ± SEM; n= animals per group. * P<0.05; ** P<0.01; *** P<0.001, ns= not significant. (T-Student’s test unpaired).

**Supplementary Figure 7. Impact of combined genetic and chemical inhibition of ERK1/2 on BRAF^V600E^-induced phenotypes in the Club cells and lung lymphocytes in placebo-treated mice.**

(**A-C**) Representative images showing double immunostainings aimed at detecting CC10 in combination with either (**A**) Ki67, (**B**) p21^CIP1^ or (**C**) γH2AX markers in lung sections of TMX-administered Erk and Erk; BRAF^V600E^ mice treated with placebo. The corresponding quantifications are shown in Figure 6G-I. (**D**) Representative images and quantifications showing SPC immunostaining in lung sections of TMX-administered Erk and Erk; BRAF^V600E^ mice treated with placebo. The corresponding quantifications are shown in Figure 6J. (**E-H**) Representative images and quantifications showing (**E**) CD45R, (**F**) CD8, (**G**) CD4 and (**H**) FOXP3 immunostainings in lung sections of TMX-administered Erk and Erk; BRAF^V600E^ mice treated with placebo. Quantifications were performed on at least five different areas of the sections in a random way. Data are expressed as mean ± SEM; n= animals per group. ns= not significant. (T-Student’s test unpaired). Arrows point to selected positive cells for the indicated marker. Insets: magnifications of areas inside dashed squares.

**Supplementary Figure 8. Impact of genetic inhibition of ERK1 on BRAF^V600E^-induced DNA damage response activation in lung lymphocytes.**

(**A-B**) Representative images and quantifications showing double immunostainings aimed at detecting γH2AX, in combination with either (**A**) CD3 or (**B**) CD45R markers in lung sections of TMX-administered Erk and Erk; BRAF^V600E^ mice in the absence of ulixertinib treatment. Quantifications were performed on at least five different areas of the sections in a random way. Data are expressed as mean ± SEM; n= animals per group. * P<0.05; ** P<0.01; *** P<0.001, **** P<0.0001; ns= not significant. (T-Student’s test unpaired). Arrows point to selected positive cells for the indicated marker. Insets: magnifications of areas inside dashed squares.

**Supplementary Figure 9. Impact of combined genetic and chemical inhibition of ERK1/2 on BRAF^V600E^-induced phenotypes in the lung parenchyma and pulmonary lymphocytes in placebo- and ulixertinib-treated mice.**

(**A-B**) Representative images and quantifications showing γH2AX immunostaining in lung sections of TMX-treated Erk and Erk;BRAF^V600E^ mice upon (**A**) placebo or (**B**) ulixertinib administration. (**C**-**D**) Representative images and quantifications showing double immunostainings aimed at detecting γH2AX, in combination with either (**C**) CD45R or (**D**) CD3 markers in lung sections of TMX-administered Erk and Erk; BRAF^V600E^ mice treated with placebo. (**E-F**) p21^CIP1^, and (**G-H**) CC3 immunostainings in lung sections of TMX-treated Erk and Erk;BRAF^V600E^ mice upon (**E**,**G**) placebo or (**F**,**H**) ulixertinib administration. Quantifications were performed on at least five different areas of the sections in a random way. Data are expressed as mean ± SEM; n= animals per group. ns= not significant. (T-Student’s test unpaired). Arrows point to selected positive cells for the indicated marker. Insets: magnifications of areas inside dashed squares.

**Supplementary Figure 10. Impact of combined genetic and chemical inhibition of ERK1/2 on BRAF^V600E^-induced phenotypes in lung lymphocytes in placebo-treated mice.**

(**A-F**). Representative images and quantifications showing double immunostainings aimed at detecting (**A,B**) Ki67, (**C,D**) p21^CIP1^ and (**E,F**) CC3 in combination with either (**A,C,E**) CD3 or (**B,D,F**) CD45R markers in lung sections of TMX-administered Erk, and Erk; BRAF^V600E^ mice treated with placebo. Quantifications were performed on at least five different areas of the sections in a random way. Data are expressed as mean ± SEM; n= animals per group. * P<0.05; ** P<0.01; *** P<0.001, **** P<0.0001; ns= not significant. (T-Student’s test unpaired). Arrows point to selected positive cells for the indicated marker. Insets: magnifications of areas inside dashed squares.
