# Supplementary material for: Differential contribution for ERK1 and ERK2 kinases in BRAFV600E-triggered phenotypes in adult mouse models
Source: Cell Death Differ. 2024 May 2;31(6):804–19. doi: 10.1038/s41418-024-01300-x (PMC11165013; doi:10.1038/s41418-024-01300-x)
Supplement: Supplementary file 2 — Supplementary Figure 1 [file 41418_2024_1300_MOESM2_ESM.pptx]

## Slide 1
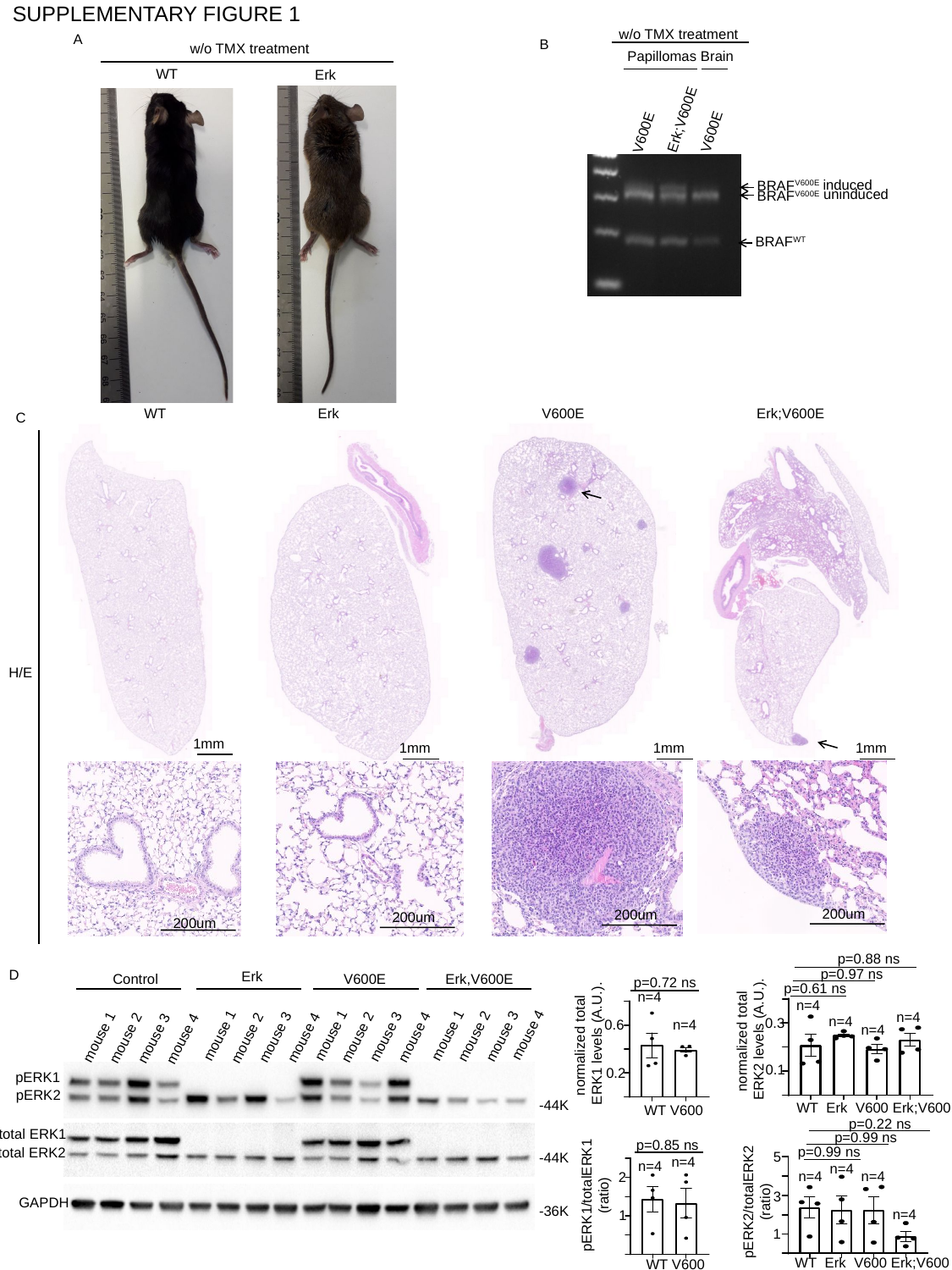

SUPPLEMENTARY FIGURE 1
w/o TMX treatment
A
B
w/o TMX treatment
Papillomas Brain
WT
Erk
Erk;V600E
V600E
V600E
induced
BRAFV600E
uninduced
BRAFV600E
BRAFWT
Erk
V600E
Erk;V600E
WT
C
H/E
1mm
1mm
1mm
1mm
200um
200um
200um
200um
p=0.88 ns
p=0.97 ns
D
Erk
Control
V600E
Erk,V600E
mouse 4
mouse 1
mouse 1
mouse 1
mouse 1
mouse 2
mouse 3
mouse 3
mouse 2
mouse 4
mouse 3
mouse 2
mouse 4
mouse 3
mouse 4
mouse 2
pERK1
pERK2
-44K
total ERK1
total ERK2
-44K
GAPDH
-36K
p=0.72 ns
p=0.61 ns
n=4
n=4
n=4
n=4
0.3
n=4
0.6
n=4
normalized total
 ERK2 levels (A.U.).
normalized total
 ERK1 levels (A.U.).
0.1
0.2
WT Erk V600 Erk;V600
WT V600
p=0.22 ns
p=0.99 ns
p=0.85 ns
p=0.99 ns
5
n=4
n=4
n=4
n=4
n=4
2
pERK1/totalERK1
(ratio)
pERK2/totalERK2
(ratio)
3
n=4
1
1
WT Erk V600 Erk;V600
WT V600
