# Supplementary material for: Differential contribution for ERK1 and ERK2 kinases in BRAFV600E-triggered phenotypes in adult mouse models
Source: Cell Death Differ. 2024 May 2;31(6):804–19. doi: 10.1038/s41418-024-01300-x (PMC11165013; doi:10.1038/s41418-024-01300-x)
Supplement: Supplementary file 3 — Supplementary Figure 2 [file 41418_2024_1300_MOESM3_ESM.pptx]

## Slide 1
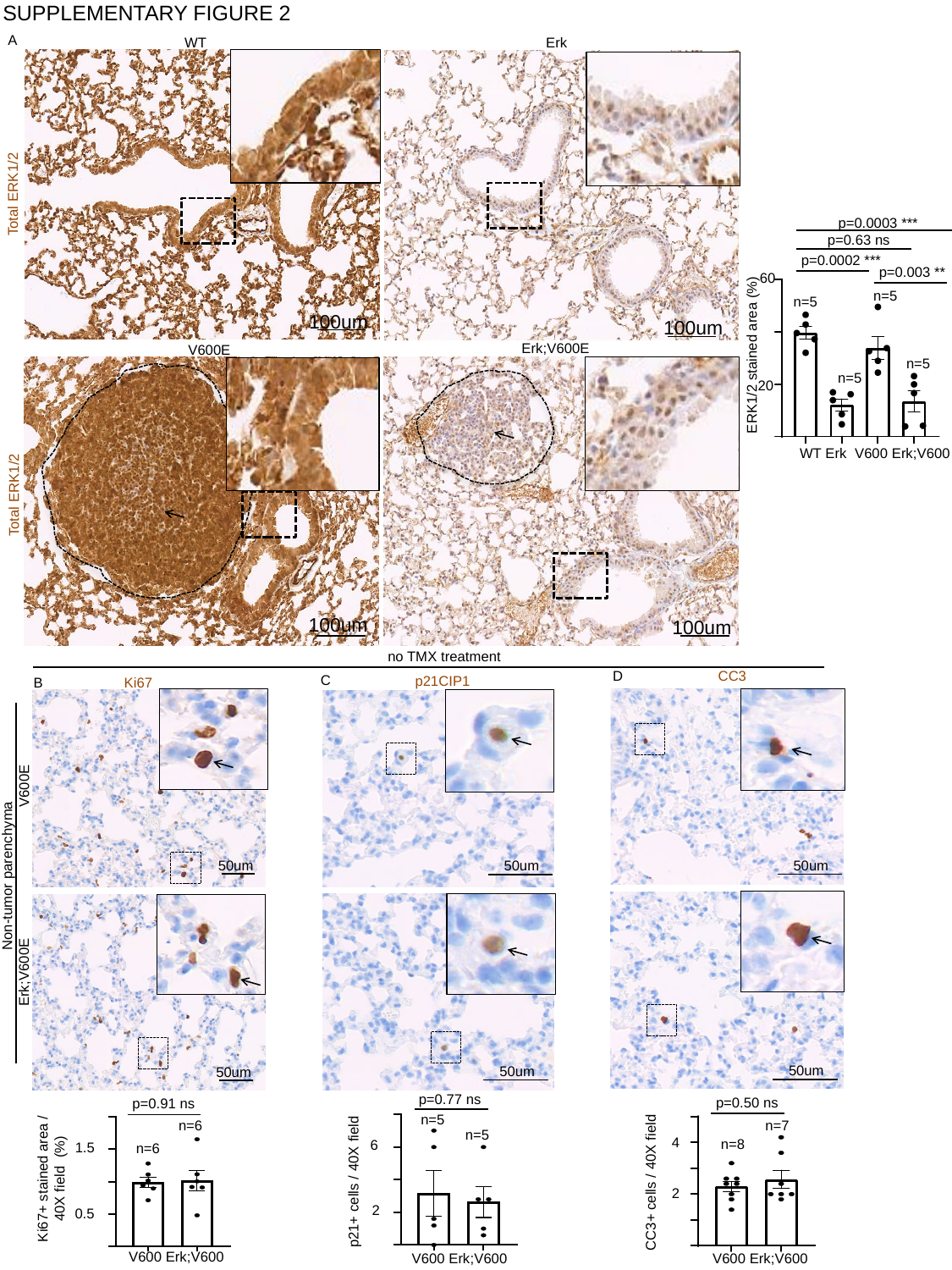

SUPPLEMENTARY FIGURE 2
A
WT
Erk
Total ERK1/2
p=0.0003 ***
p=0.63 ns
p=0.0002 ***
p=0.003 **
60
n=5
n=5
100um
100um
ERK1/2 stained area (%)
Erk;V600E
V600E
n=5
n=5
20
WT Erk V600 Erk;V600
Total ERK1/2
100um
100um
no TMX treatment
D
CC3
C
p21CIP1
B
Ki67
V600E
Non-tumor parenchyma
50um
50um
50um
Erk;V600E
50um
50um
50um
p=0.77 ns
p=0.50 ns
p=0.91 ns
n=5
n=7
n=6
n=5
4
n=8
6
1.5
n=6
Ki67+ stained area / 40X field (%)
p21+ cells / 40X field
CC3+ cells / 40X field
2
2
0.5
V600 Erk;V600
V600 Erk;V600
V600 Erk;V600
