# Supplementary material for: Differential contribution for ERK1 and ERK2 kinases in BRAFV600E-triggered phenotypes in adult mouse models
Source: Cell Death Differ. 2024 May 2;31(6):804–19. doi: 10.1038/s41418-024-01300-x (PMC11165013; doi:10.1038/s41418-024-01300-x)
Supplement: Supplementary file 4 — Supplementary Figure 3 [file 41418_2024_1300_MOESM4_ESM.pptx]

## Slide 1
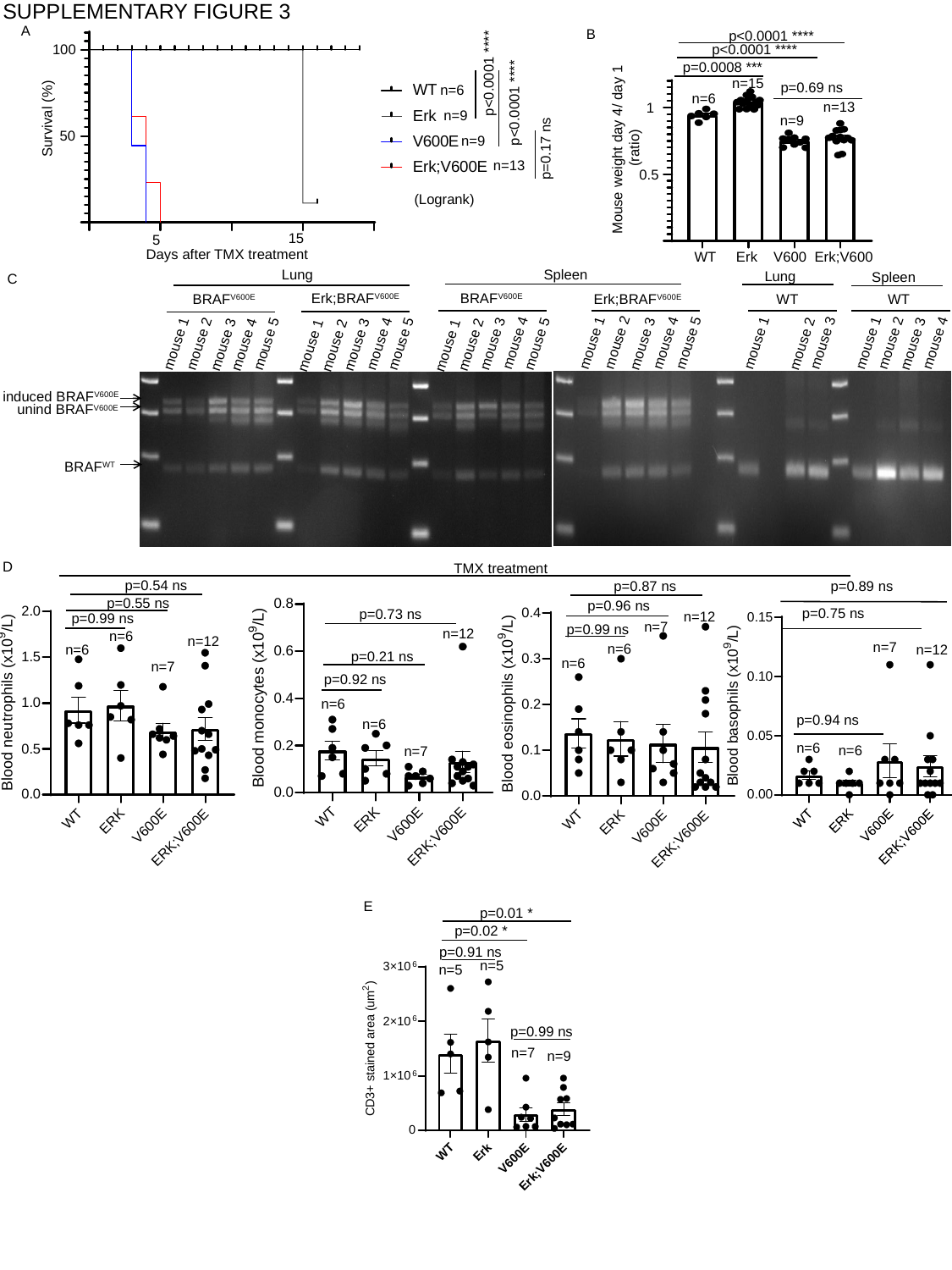

SUPPLEMENTARY FIGURE 3
A
B
p<0.0001 ****
100
p<0.0001 ****
p=0.0008 ***
p<0.0001 ****
n=15
p=0.69 ns
n=6
n=6
p<0.0001 ****
n=13
1
n=9
Survival (%)
n=9
p=0.17 ns
50
Mouse weight day 4/ day 1
(ratio)
n=9
n=13
0.5
(Logrank)
15
5
Days after TMX treatment
WT Erk V600 Erk;V600
Lung
Spleen
Lung
Spleen
C
Erk;BRAFV600E
BRAFV600E
BRAFV600E
Erk;BRAFV600E
WT
WT
mouse 2
mouse 5
mouse 1
mouse 1
mouse 1
mouse 2
mouse 4
mouse 4
mouse 3
mouse 4
mouse 3
mouse 3
mouse 1
mouse 3
mouse 2
mouse 5
mouse 4
mouse 2
mouse 5
mouse 5
mouse 4
mouse 3
mouse 2
mouse 3
mouse 2
mouse 1
mouse 1
induced BRAFV600E
unind BRAFV600E
BRAFWT
D
TMX treatment
p=0.54 ns
p=0.87 ns
p=0.89 ns
p=0.55 ns
p=0.96 ns
p=0.75 ns
p=0.73 ns
n=12
p=0.99 ns
n=7
p=0.99 ns
n=12
n=6
n=12
n=7
n=6
n=6
n=12
p=0.21 ns
n=6
n=7
p=0.92 ns
n=6
p=0.94 ns
n=6
n=6
n=6
n=7
E
p=0.01 *
p=0.02 *
p=0.91 ns
n=5
n=5
p=0.99 ns
n=7
n=9
