# Supplementary material for: Differential contribution for ERK1 and ERK2 kinases in BRAFV600E-triggered phenotypes in adult mouse models
Source: Cell Death Differ. 2024 May 2;31(6):804–19. doi: 10.1038/s41418-024-01300-x (PMC11165013; doi:10.1038/s41418-024-01300-x)
Supplement: Supplementary file 5 — Supplementary Figure 4 [file 41418_2024_1300_MOESM5_ESM.pptx]

## Slide 1
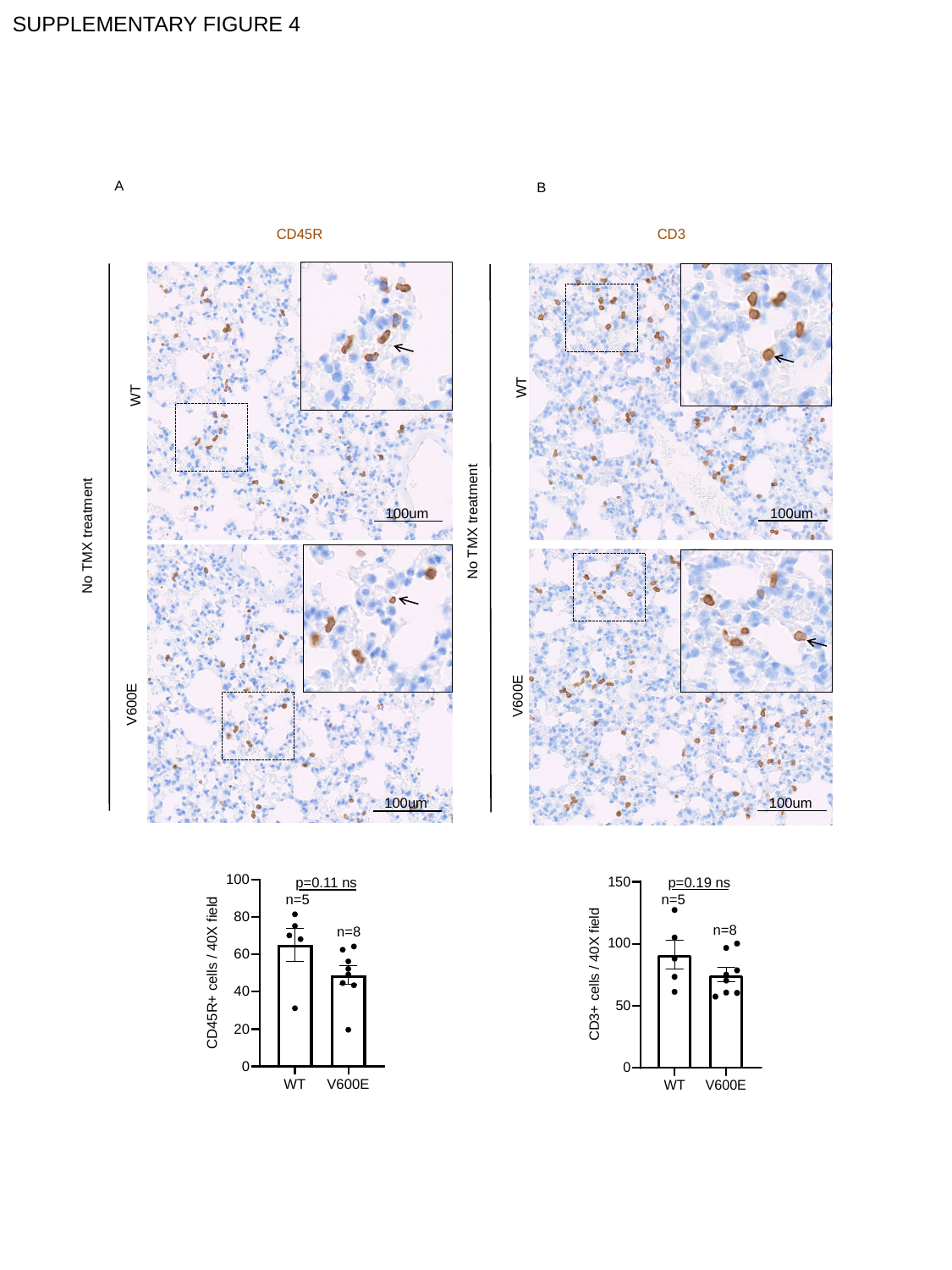

SUPPLEMENTARY FIGURE 4
A
B
CD45R
CD3
WT
WT
No TMX treatment
No TMX treatment
100um
100um
V600E
V600E
100um
100um
p=0.19 ns
p=0.11 ns
n=5
n=5
n=8
n=8
