# Supplementary material for: Differential contribution for ERK1 and ERK2 kinases in BRAFV600E-triggered phenotypes in adult mouse models
Source: Cell Death Differ. 2024 May 2;31(6):804–19. doi: 10.1038/s41418-024-01300-x (PMC11165013; doi:10.1038/s41418-024-01300-x)
Supplement: Supplementary file 7 — Supplementary Figure 6 [file 41418_2024_1300_MOESM7_ESM.pptx]

## Slide 1
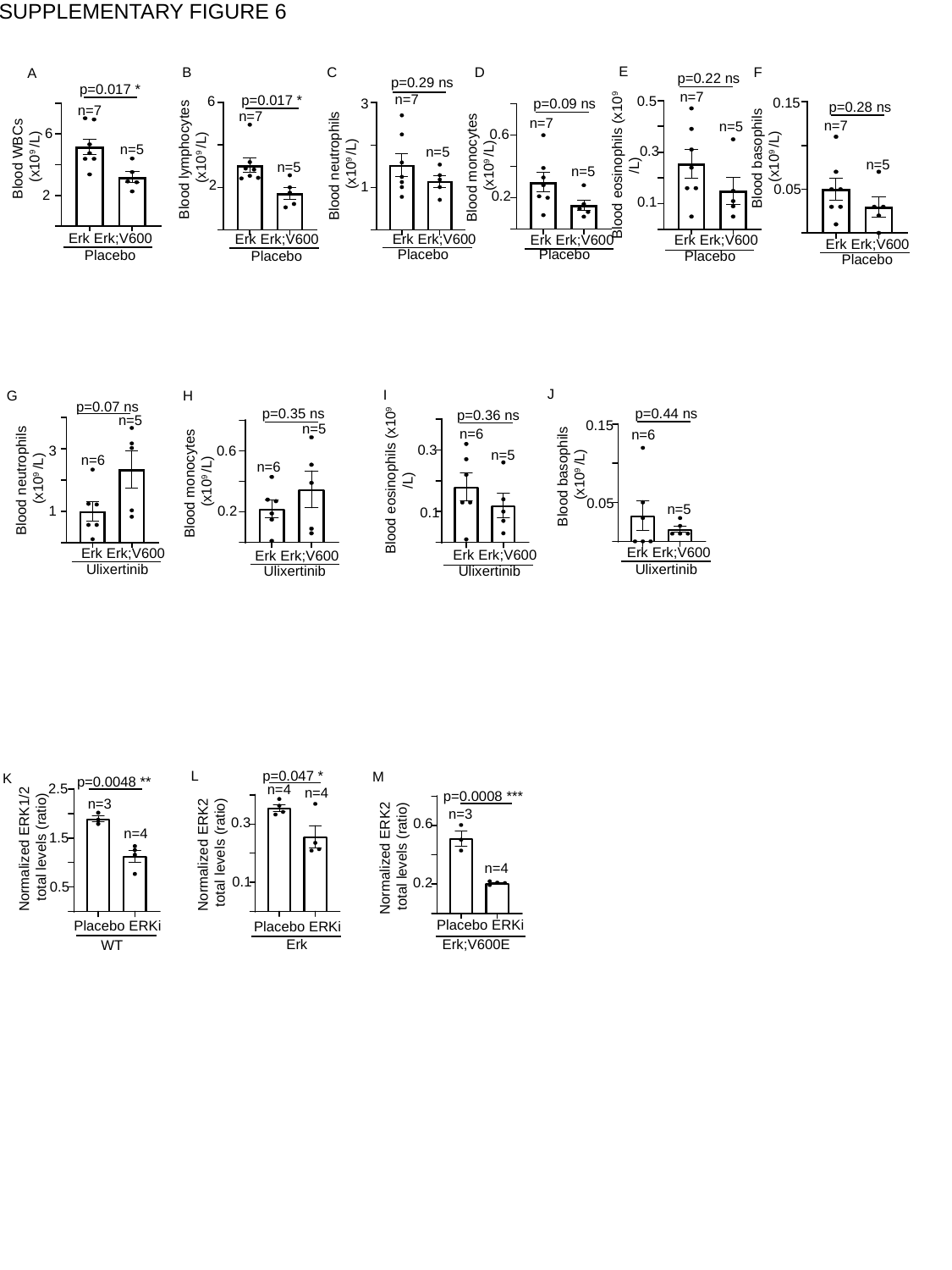

SUPPLEMENTARY FIGURE 6
E
C
F
B
D
A
p=0.22 ns
p=0.29 ns
p=0.017 *
n=7
n=7
p=0.017 *
0.5
6
0.15
3
p=0.09 ns
p=0.28 ns
n=7
n=7
n=7
n=7
n=5
6
0.6
Blood basophils
(x109 /L)
Blood WBCs
(x109 /L)
Blood lymphocytes
(x109 /L)
n=5
0.3
n=5
Blood neutrophils
(x109 /L)
Blood eosinophils (x109 /L)
Blood monocytes
(x109 /L)
n=5
n=5
n=5
2
1
0.05
2
0.2
0.1
Erk Erk;V600
Erk Erk;V600
Erk Erk;V600
Erk Erk;V600
Erk Erk;V600
Erk Erk;V600
Placebo
Placebo
Placebo
Placebo
Placebo
Placebo
J
I
G
H
p=0.07 ns
p=0.44 ns
p=0.35 ns
n=5
0.6
n=6
0.2
p=0.36 ns
n=5
0.15
n=6
n=6
0.3
3
n=5
n=6
Blood basophils
(x109 /L)
Blood neutrophils
(x109 /L)
Blood eosinophils (x109 /L)
Blood monocytes
(x109 /L)
0.05
n=5
1
0.1
Erk Erk;V600
Erk Erk;V600
Erk Erk;V600
Erk Erk;V600
Ulixertinib
Ulixertinib
Ulixertinib
Ulixertinib
p=0.0008 ***
n=3
0.6
Normalized ERK2
total levels (ratio)
n=4
0.2
Placebo ERKi
Erk;V600E
p=0.047 *
L
M
K
p=0.0048 **
2.5
n=4
n=4
n=3
0.3
n=4
1.5
Normalized ERK1/2
total levels (ratio)
Normalized ERK2
total levels (ratio)
0.1
0.5
Placebo ERKi
Placebo ERKi
Erk
WT
