# Supplementary material for: Differential contribution for ERK1 and ERK2 kinases in BRAFV600E-triggered phenotypes in adult mouse models
Source: Cell Death Differ. 2024 May 2;31(6):804–19. doi: 10.1038/s41418-024-01300-x (PMC11165013; doi:10.1038/s41418-024-01300-x)
Supplement: Supplementary file 8 — Supplementary Figure 7 [file 41418_2024_1300_MOESM8_ESM.pptx]

## Slide 1
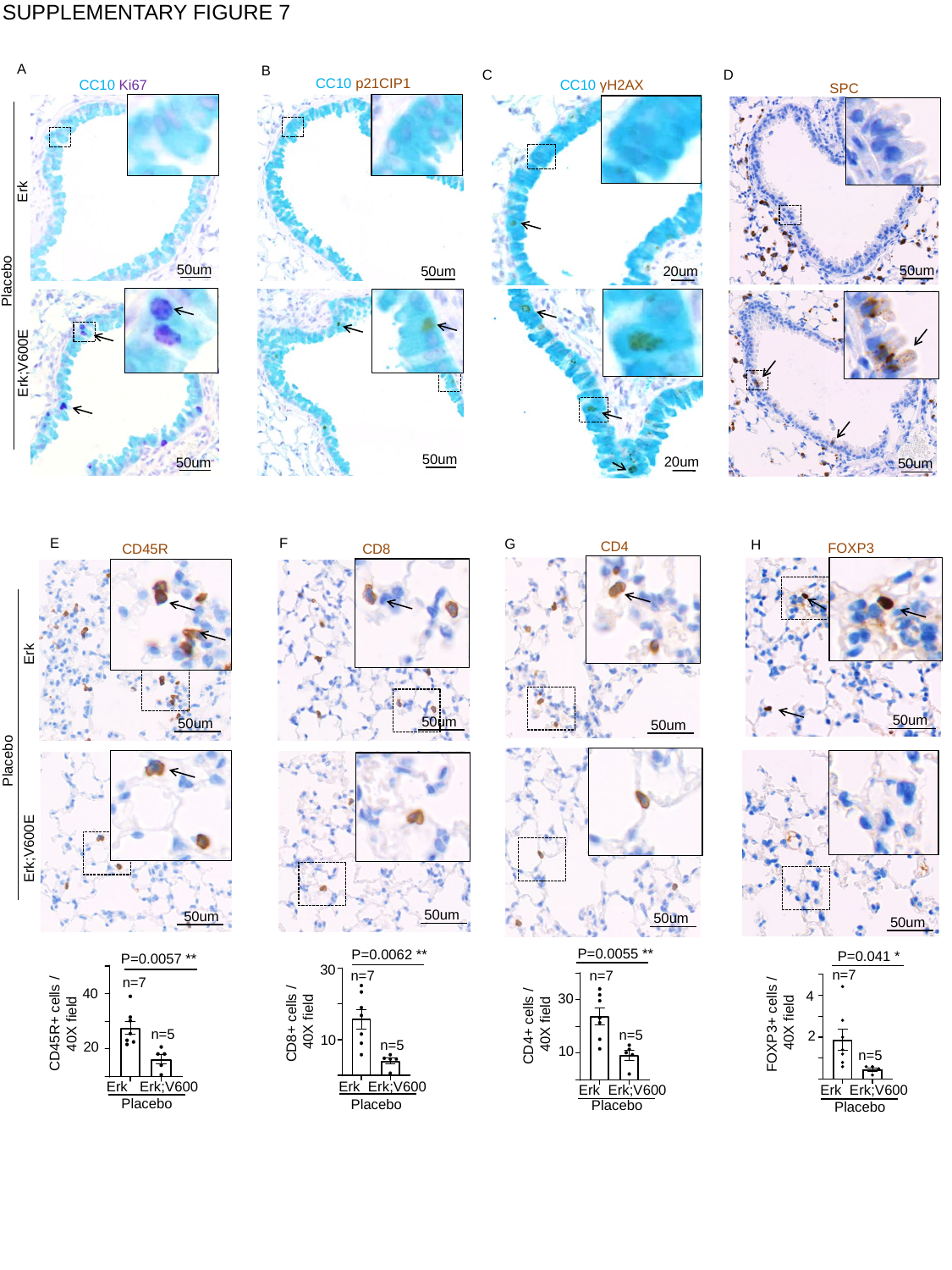

SUPPLEMENTARY FIGURE 7
A
B
C
D
CC10 p21CIP1
CC10 Ki67
CC10 γH2AX
SPC
Erk
50um
50um
20um
50um
Placebo
Erk;V600E
50um
20um
50um
50um
E
F
G
H
CD4
FOXP3
CD45R
CD8
Erk
Placebo
50um
50um
50um
50um
Erk;V600E
50um
50um
50um
50um
P=0.0055 **
P=0.0062 **
P=0.041 *
P=0.0057 **
30
n=7
n=7
n=7
n=7
40
4
30
CD8+ cells /
40X field
FOXP3+ cells /
40X field
CD45R+ cells /
40X field
CD4+ cells /
40X field
n=5
n=5
2
10
n=5
20
10
n=5
 Erk Erk;V600
 Erk Erk;V600
Erk Erk;V600
 Erk Erk;V600
Placebo
Placebo
Placebo
Placebo
