# Supplementary material for: Differential contribution for ERK1 and ERK2 kinases in BRAFV600E-triggered phenotypes in adult mouse models
Source: Cell Death Differ. 2024 May 2;31(6):804–19. doi: 10.1038/s41418-024-01300-x (PMC11165013; doi:10.1038/s41418-024-01300-x)
Supplement: Supplementary file 9 — Supplementary Figure 8 [file 41418_2024_1300_MOESM9_ESM.pptx]

## Slide 1
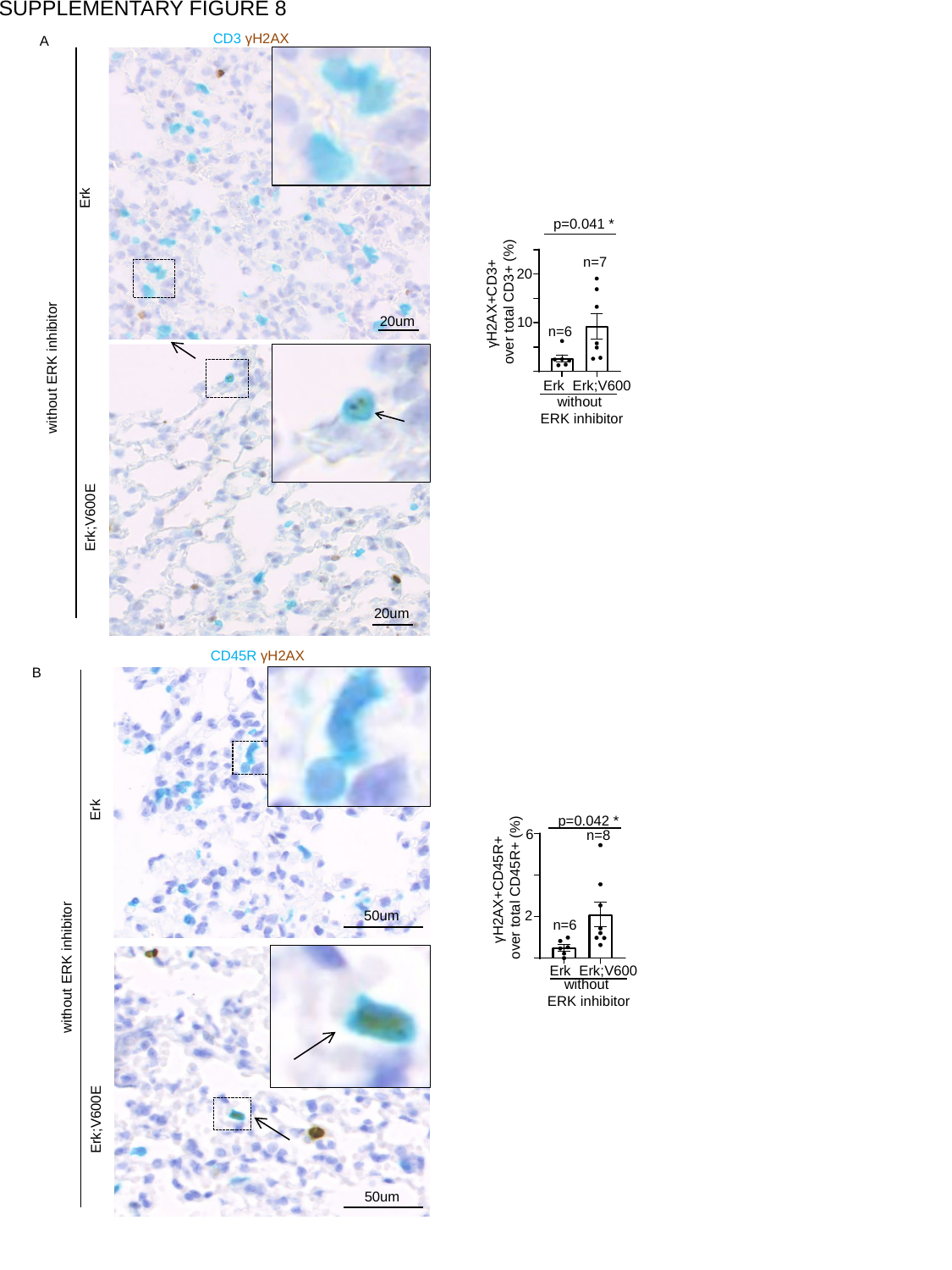

SUPPLEMENTARY FIGURE 8
CD3 γH2AX
A
Erk
without ERK inhibitor
20um
Erk;V600E
20um
p=0.041 *
n=7
20
γH2AX+CD3+
over total CD3+ (%)
10
n=6
 Erk Erk;V600
without
ERK inhibitor
CD45R γH2AX
B
50um
50um
Erk
without ERK inhibitor
20um
Erk;V600E
p=0.042 *
6
n=8
γH2AX+CD45R+
over total CD45R+ (%)
2
n=6
 Erk Erk;V600
without
ERK inhibitor
