# Supplementary material for: Differential contribution for ERK1 and ERK2 kinases in BRAFV600E-triggered phenotypes in adult mouse models
Source: Cell Death Differ. 2024 May 2;31(6):804–19. doi: 10.1038/s41418-024-01300-x (PMC11165013; doi:10.1038/s41418-024-01300-x)
Supplement: Supplementary file 10 — Supplementary Figure 9 [file 41418_2024_1300_MOESM10_ESM.pptx]

## Slide 1
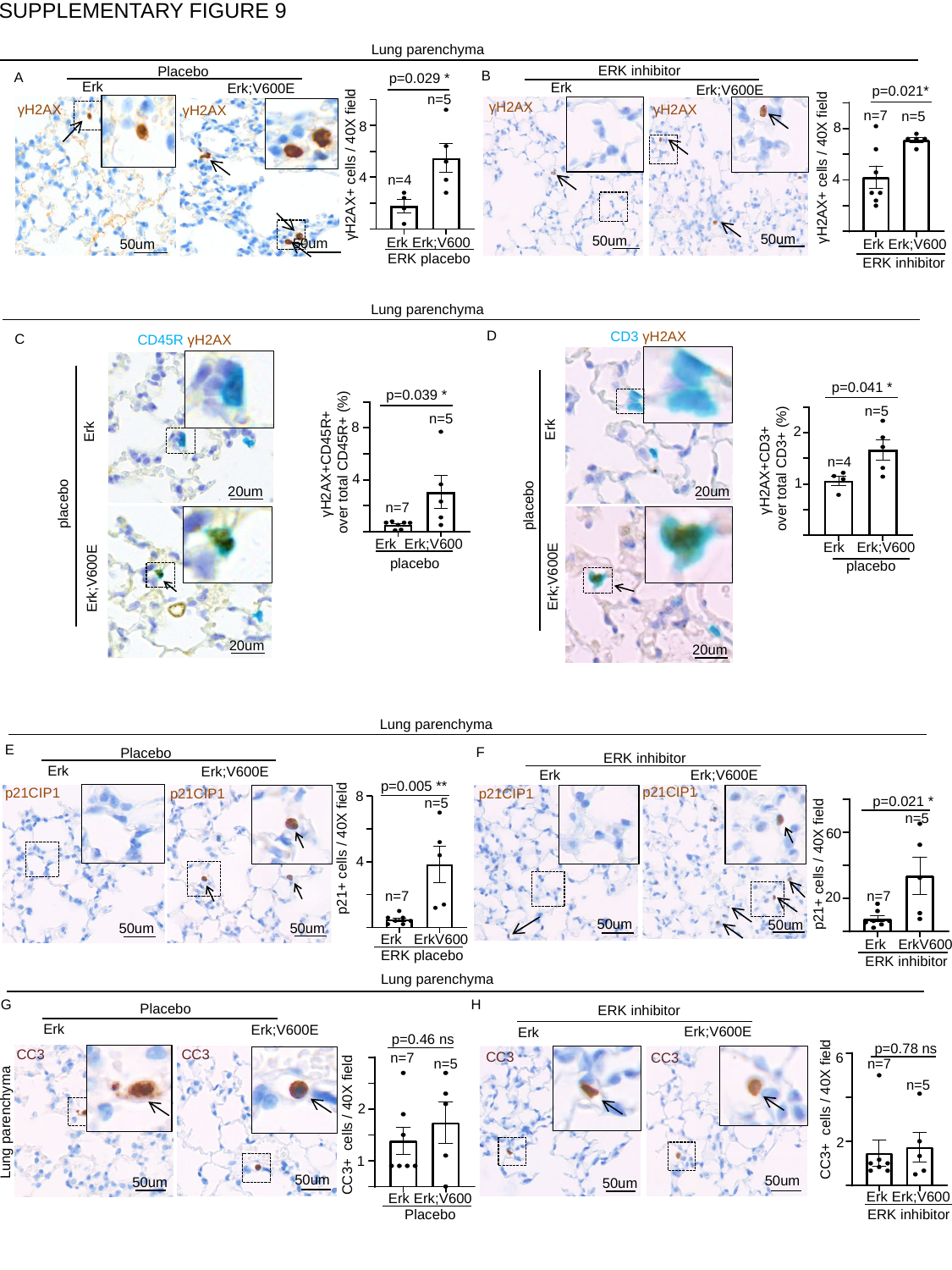

SUPPLEMENTARY FIGURE 9
Lung parenchyma
ERK inhibitor
Placebo
B
A
p=0.029 *
Erk
Erk
Erk;V600E
Erk;V600E
p=0.021*
n=5
γH2AX
γH2AX
γH2AX
γH2AX
n=7
n=5
8
8
γH2AX+ cells / 40X field
γH2AX+ cells / 40X field
4
4
n=4
50um
50um
Erk Erk;V600
50um
Erk Erk;V600
50um
ERK placebo
ERK inhibitor
Lung parenchyma
D
CD3 γH2AX
C
CD45R γH2AX
p=0.041 *
p=0.039 *
n=5
Erk
Erk
n=5
8
2
γH2AX+CD45R+
over total CD45R+ (%)
γH2AX+CD3+
over total CD3+ (%)
n=4
placebo
placebo
4
1
20um
20um
n=7
 Erk Erk;V600
 Erk Erk;V600
placebo
placebo
Erk;V600E
Erk;V600E
20um
20um
Lung parenchyma
E
F
Placebo
ERK inhibitor
Erk
Erk;V600E
Erk;V600E
Erk
p=0.005 **
p21CIP1
p21CIP1
p21CIP1
p21CIP1
p21CIP1
8
p=0.021 *
n=5
n=5
60
p21+ cells / 40X field
4
p21+ cells / 40X field
n=7
n=7
20
50um
50um
50um
50um
Erk ErkV600
Erk ErkV600
ERK placebo
ERK inhibitor
Lung parenchyma
G
H
Placebo
ERK inhibitor
Erk
Erk;V600E
Erk;V600E
Erk
p=0.46 ns
p=0.78 ns
CC3
CC3
CC3
6
CC3
n=7
n=7
n=5
n=5
2
CC3+ cells / 40X field
Lung parenchyma
CC3+ cells / 40X field
2
1
50um
50um
50um
50um
Erk Erk;V600
Erk Erk;V600
ERK inhibitor
Placebo
