# Supplementary material for: Differential contribution for ERK1 and ERK2 kinases in BRAFV600E-triggered phenotypes in adult mouse models
Source: Cell Death Differ. 2024 May 2;31(6):804–19. doi: 10.1038/s41418-024-01300-x (PMC11165013; doi:10.1038/s41418-024-01300-x)
Supplement: Supplementary file 11 — Supplementary Figure 10 [file 41418_2024_1300_MOESM11_ESM.pptx]

## Slide 1
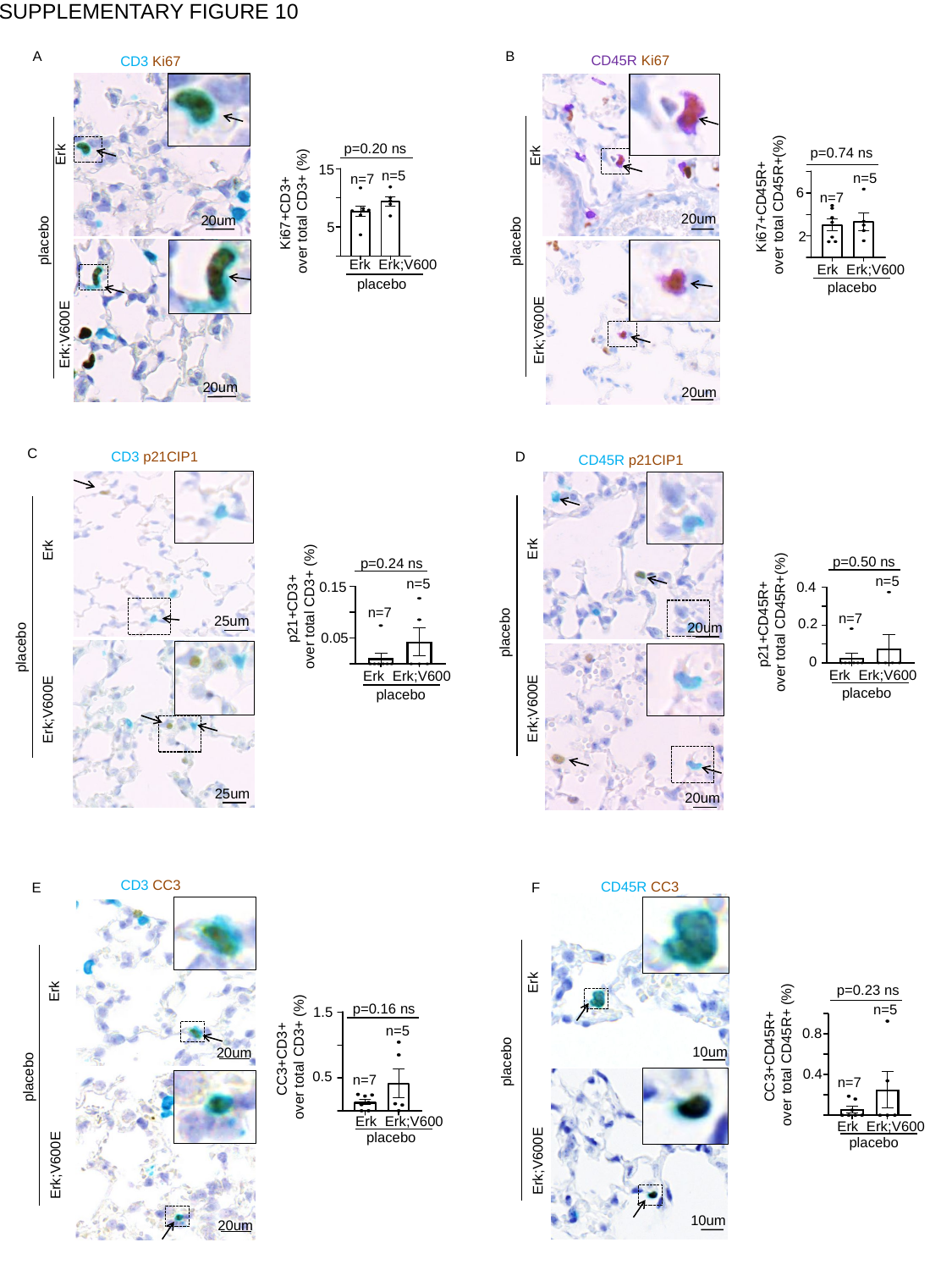

SUPPLEMENTARY FIGURE 10
A
B
CD45R Ki67
CD3 Ki67
Erk
Erk
p=0.20 ns
p=0.74 ns
15
n=5
n=5
n=7
6
Ki67+CD45R+
over total CD45R+(%)
n=7
Ki67+CD3+
over total CD3+ (%)
20um
20um
5
placebo
2
placebo
 Erk Erk;V600
Erk Erk;V600
placebo
placebo
Erk;V600E
Erk;V600E
20um
20um
C
D
CD3 p21CIP1
CD45R p21CIP1
Erk
Erk
p=0.50 ns
p=0.24 ns
n=5
n=5
0.15
0.4
p21+CD3+
over total CD3+ (%)
placebo
20um
n=7
placebo
p21+CD45R+
over total CD45R+(%)
n=7
25um
0.2
20um
0.05
0
 Erk Erk;V600
 Erk Erk;V600
placebo
placebo
Erk;V600E
Erk;V600E
25um
20um
CD3 CC3
CD45R CC3
F
E
Erk
Erk
p=0.23 ns
p=0.16 ns
1.5
n=5
CC3+CD3+
over total CD3+ (%)
0.5
n=7
Erk Erk;V600
placebo
n=5
placebo
0.8
placebo
CC3+CD45R+
over total CD45R+ (%)
10um
20um
0.4
n=7
Erk Erk;V600
Erk;V600E
placebo
Erk;V600E
Erk;V600E
10um
20um
