# Supplementary material for: Differential contribution for ERK1 and ERK2 kinases in BRAFV600E-triggered phenotypes in adult mouse models
Source: Cell Death Differ. 2024 May 2;31(6):804–19. doi: 10.1038/s41418-024-01300-x (PMC11165013; doi:10.1038/s41418-024-01300-x)

FIGURE 1D

gapdh

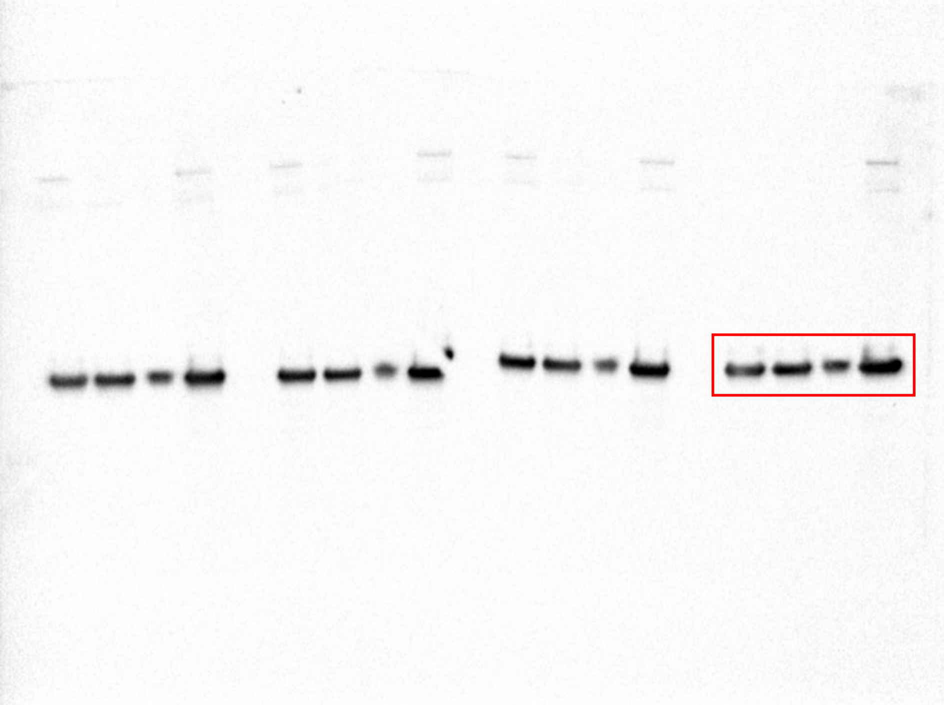

Total erk

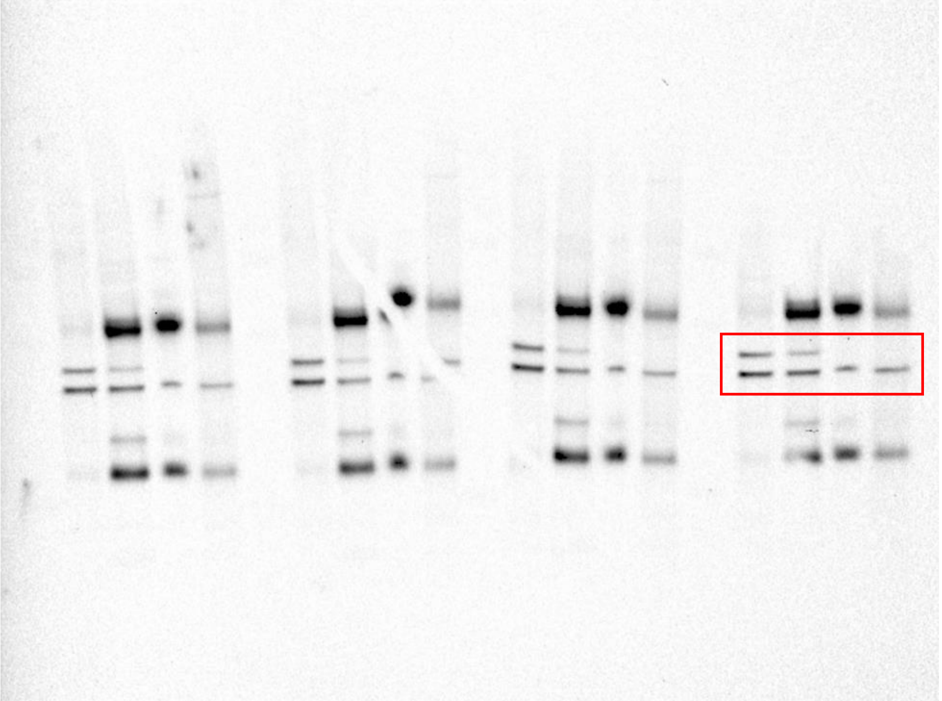

Phospho erk

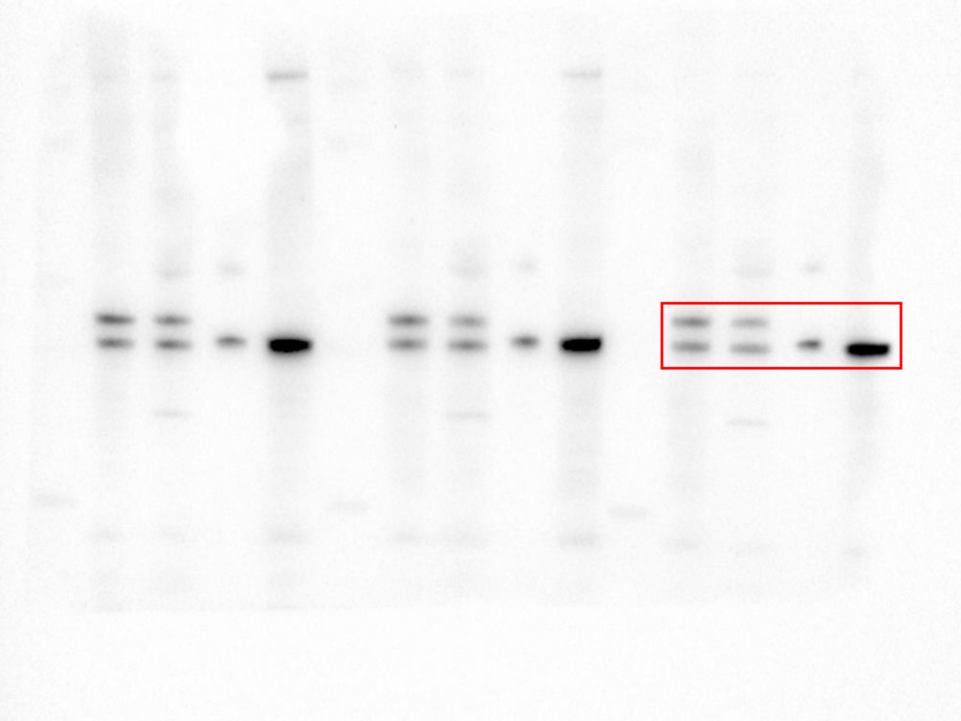

FIGURE 3A

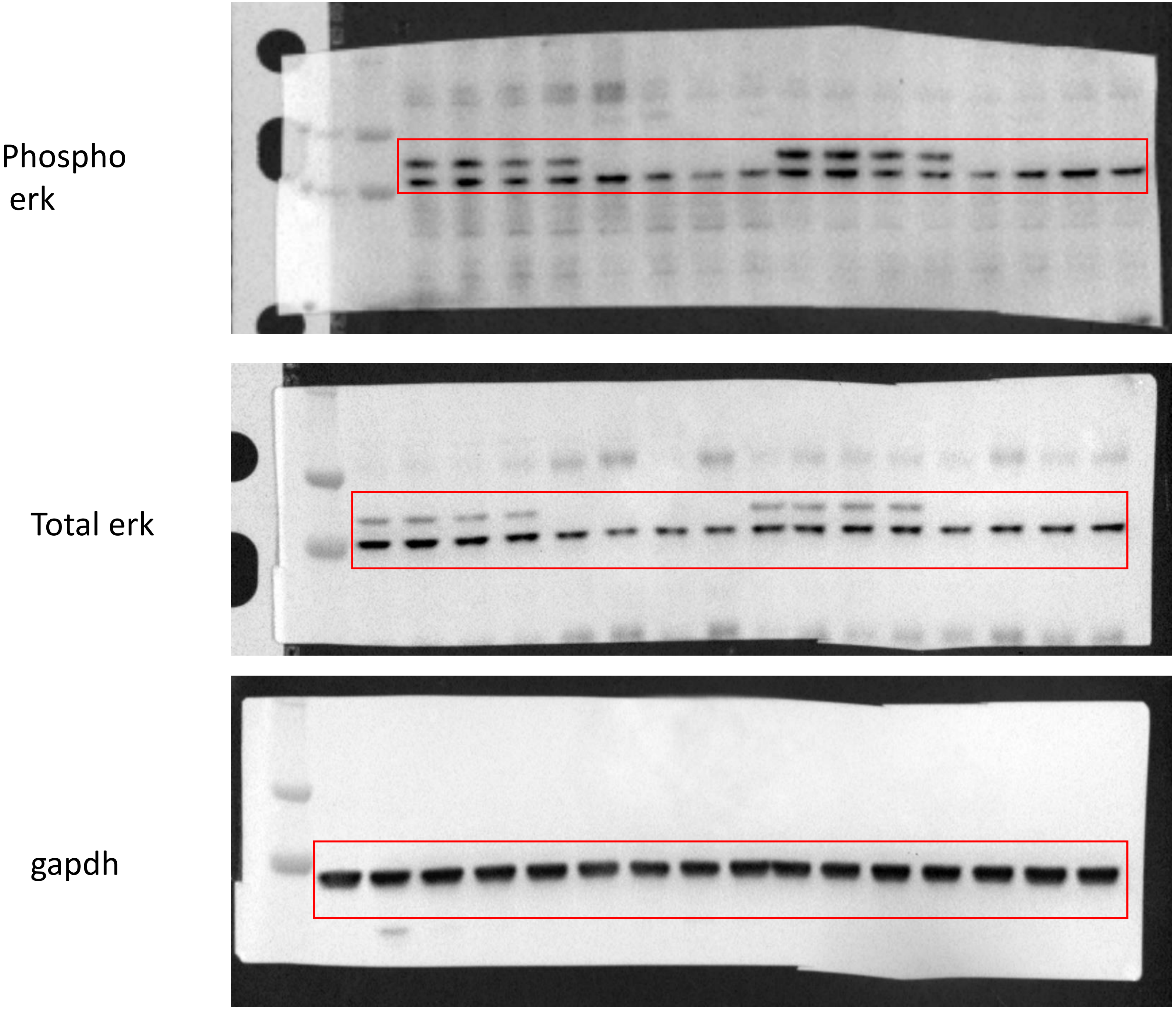

FIGURE 5A

gapdh

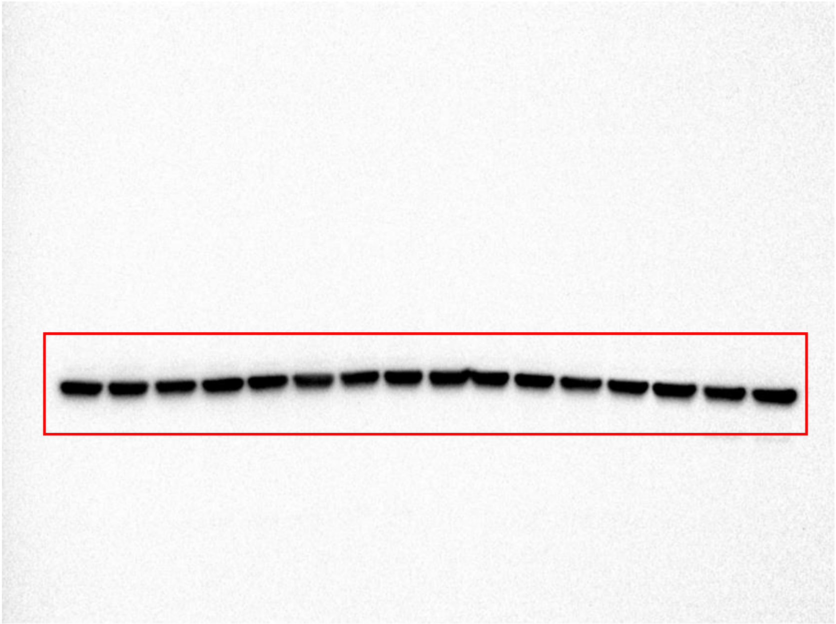

Phospho erk

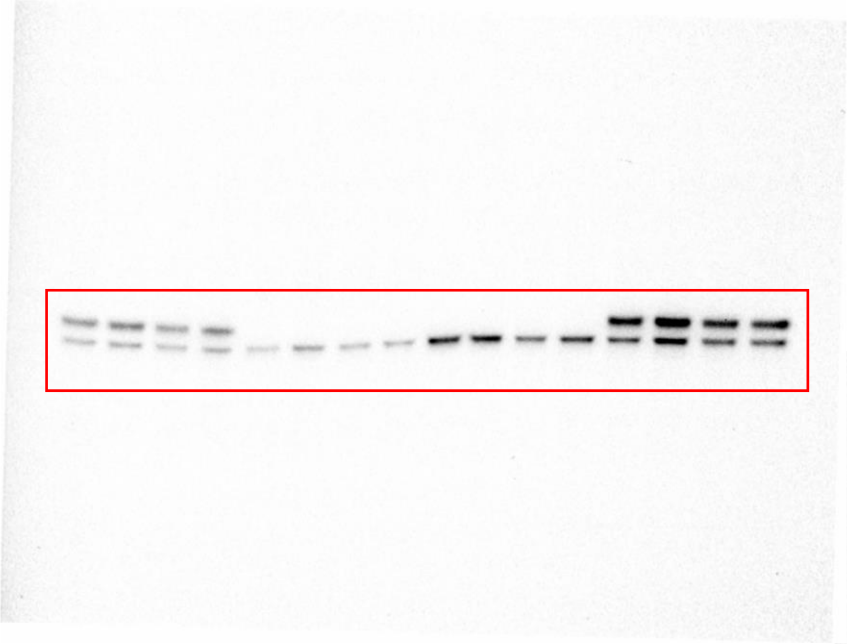

Total erk

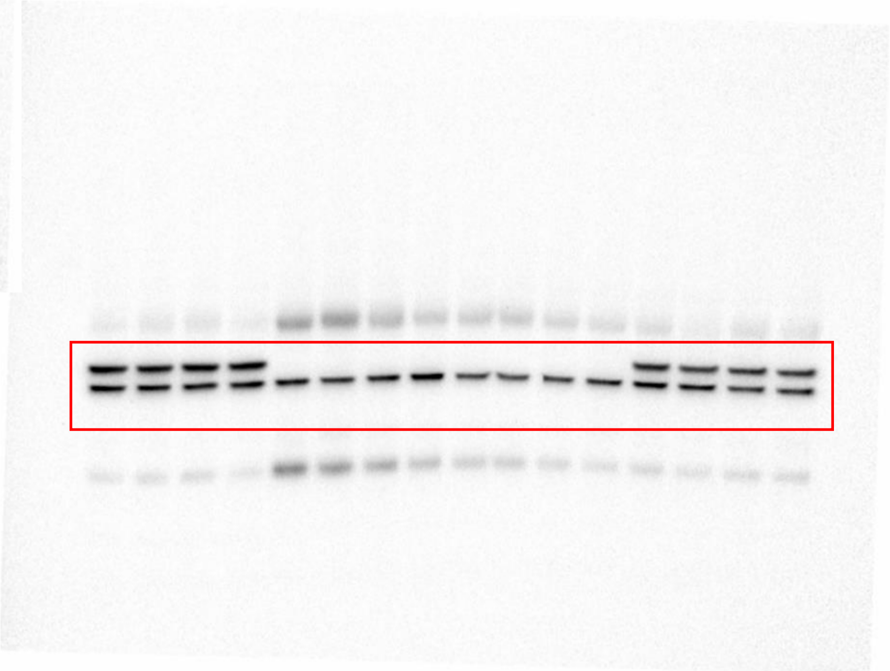

FIGURE 6D

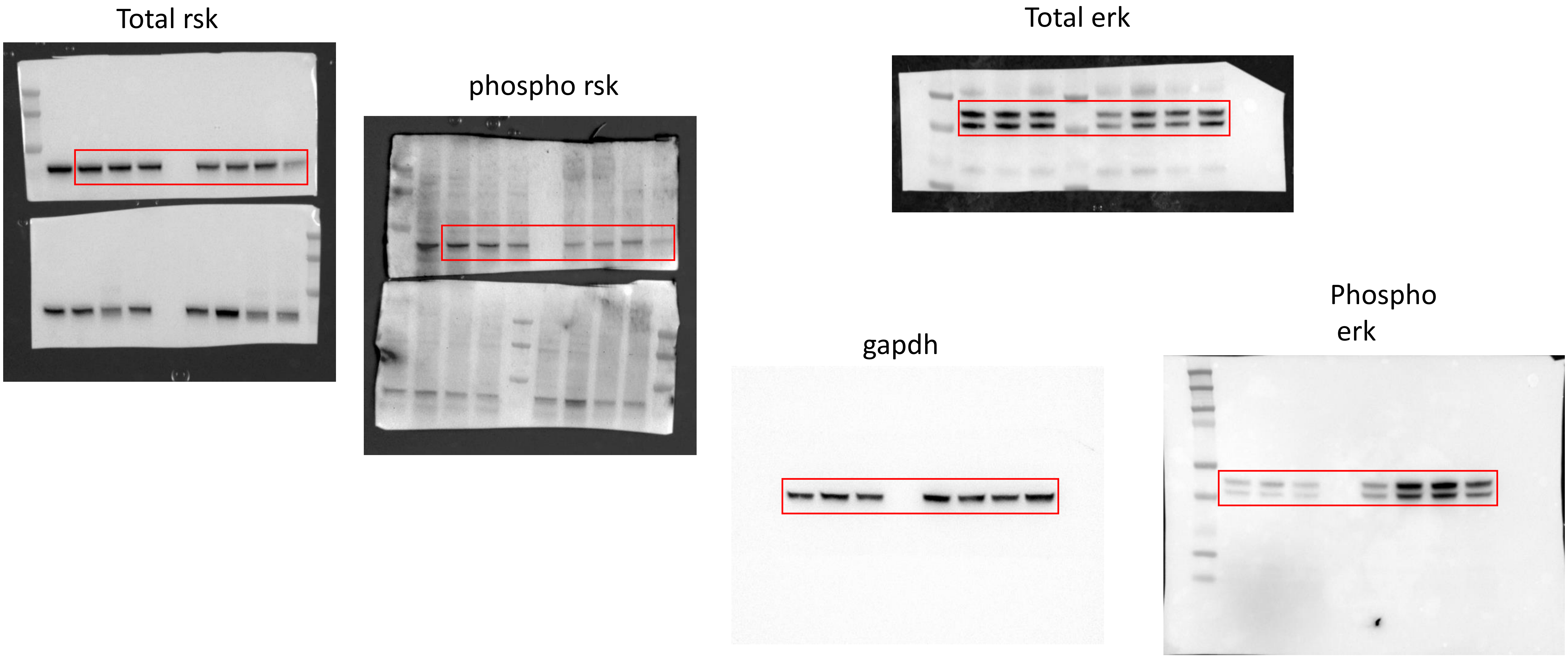

FIGURE 6E

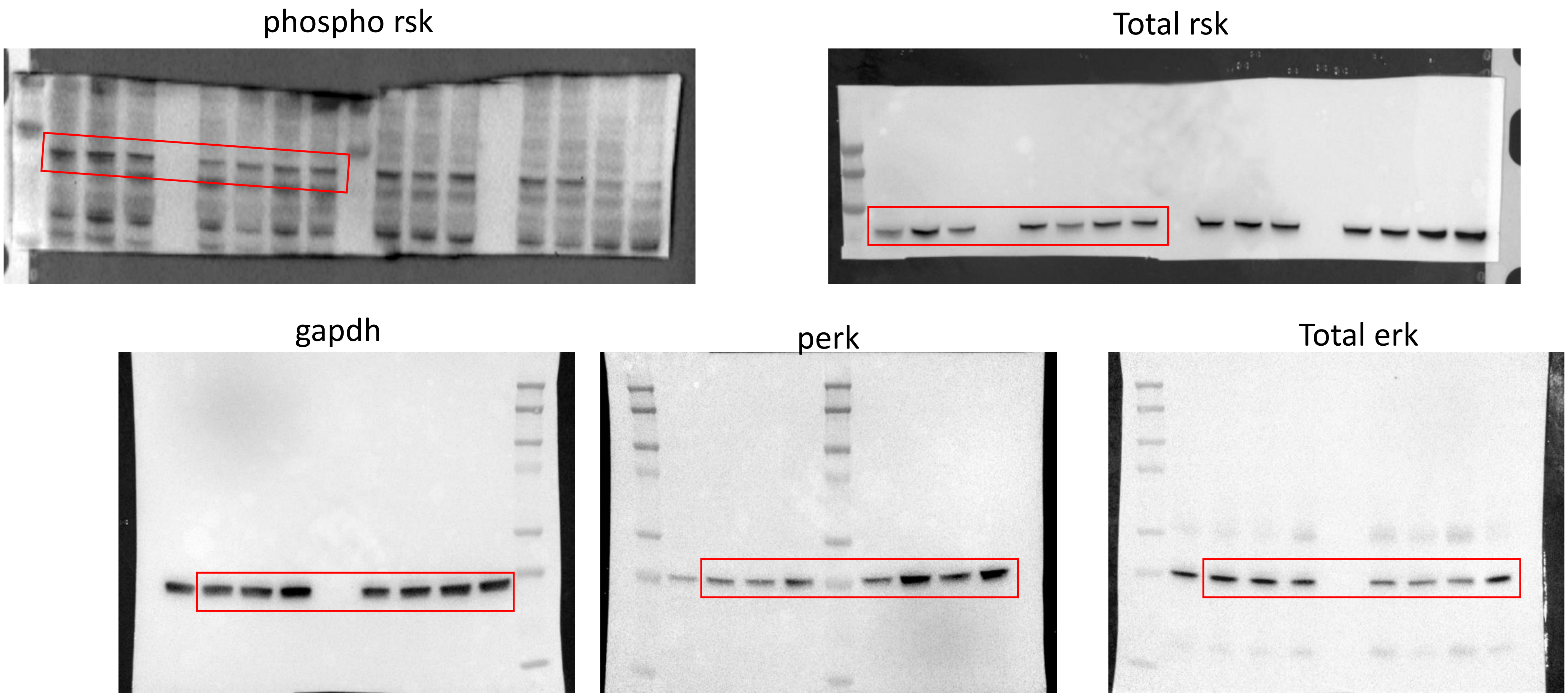

FIGURE 6F

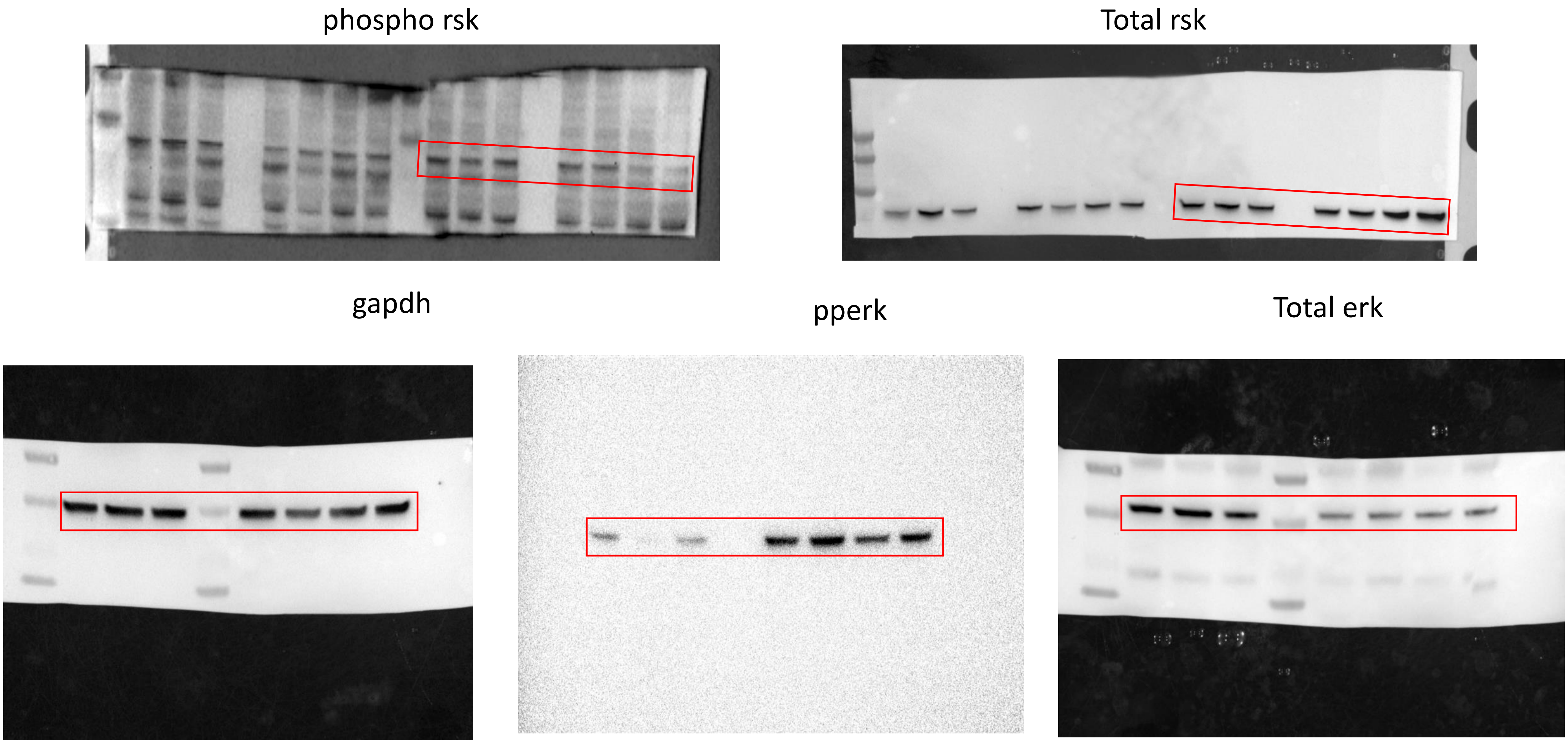

SUPPLEMENTARY FIGURE 1D

gapdh

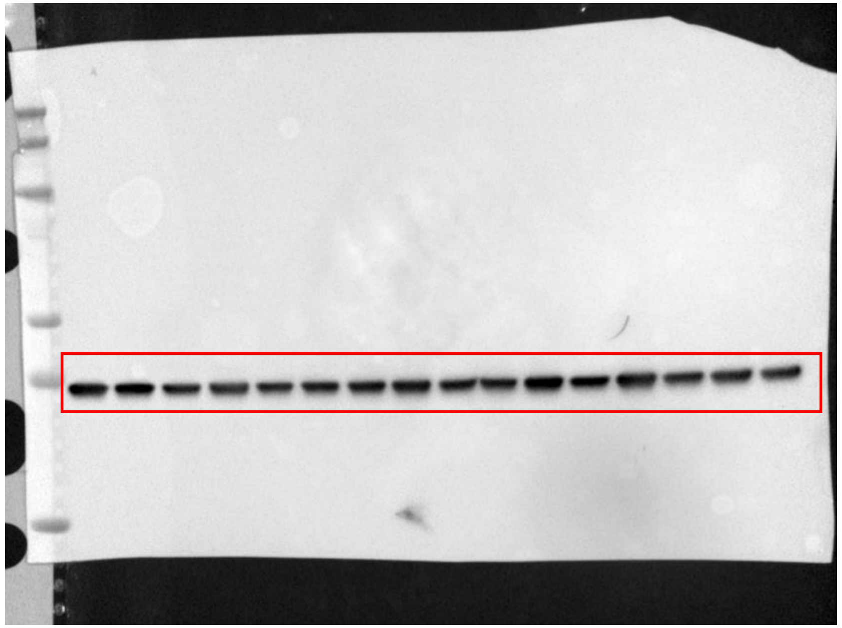

Phospho erk

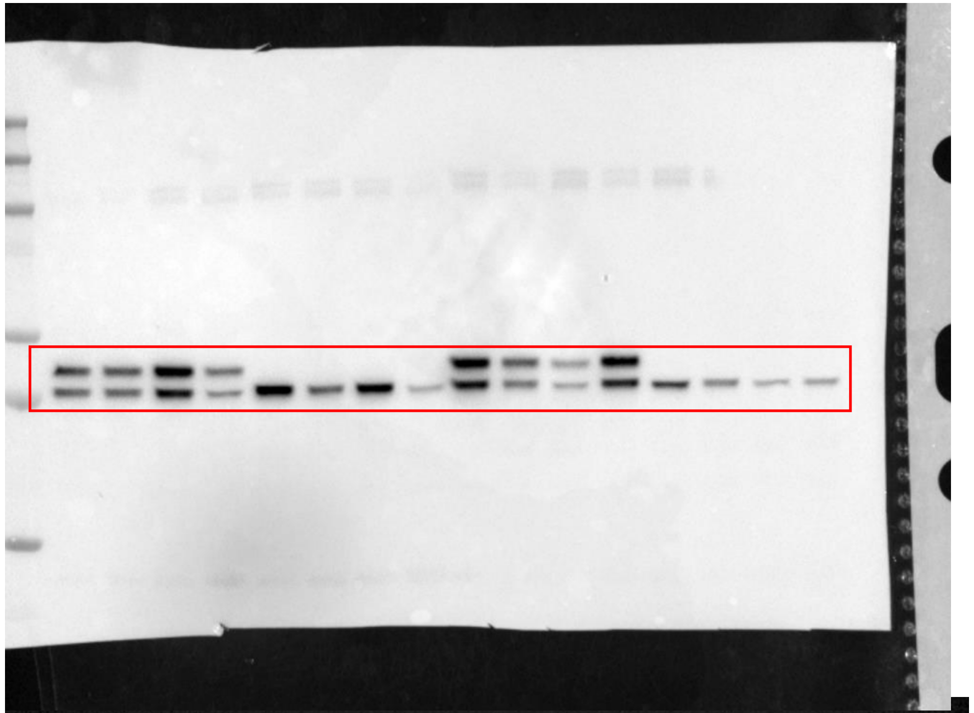

Total erk

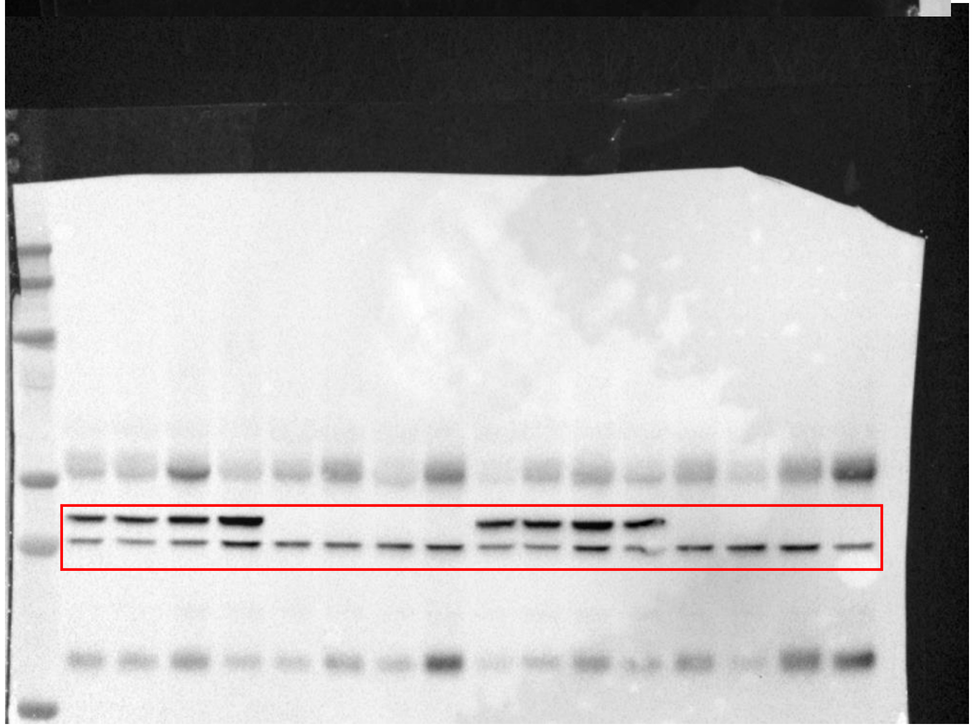

Supplement: Supplementary file 12 — Uncropped WBs [file 41418_2024_1300_MOESM12_ESM.pdf]
